# Sec16 and Sed4 interdependently function as interaction and localization partners at ER exit sites

**DOI:** 10.1242/jcs.261094

**Published:** 2023-05-09

**Authors:** Tomohiro Yorimitsu, Ken Sato

**Affiliations:** Department of Life Sciences, Graduate School of Arts and Sciences, University of Tokyo, Tokyo 153-8902, Japan

**Keywords:** Endoplasmic reticulum, ER, COPII, ER exit sites, Sec16, Sed4

## Abstract

COPII proteins assemble at ER exit sites (ERES) to form transport carriers. The initiation of COPII assembly in the yeast *Saccharomyces cerevisiae* is triggered by the ER membrane protein Sec12. Sec16, which plays a critical role in COPII organization, localizes to ERES independently of Sec12. However, the mechanism underlying Sec16 localization is poorly understood. Here, we show that a Sec12 homolog, Sed4, is concentrated at ERES and mediates ERES localization of Sec16. We found that the interaction between Sec16 and Sed4 ensures their correct localization to ERES. Loss of the interaction with Sec16 leads to redistribution of Sed4 from the ERES specifically to high-curvature ER areas, such as the tubules and edges of the sheets. The luminal domain of Sed4 mediates this distribution, which is required for Sed4, but not for Sec16, to be concentrated at ERES. We further show that the luminal domain and its O-mannosylation are involved in the self-interaction of Sed4. Our findings provide insight into how Sec16 and Sed4 function interdependently at ERES.

## Teaser

**Summary:** The Sec12 homolog Sed4 interacts with Sec16 and acts to localize Sec16 to ER exit sites. The ER luminal domain of Sed4 is involved in the self-interaction and localization to the highly curved ER domains.

## INTRODUCTION

Secretory proteins are synthesized in the endoplasmic reticulum (ER) and exported to the Golgi. COPII transport carriers are sculpted from the ER membrane at ER exit sites (ERES), where COPII proteins assemble and mediate ER-to-Golgi trafficking. In *Saccharomyces cerevisiae*, seven essential COPII proteins have been identified as functioning in defining the ERES, forming COPII carrier vesicles, and loading cargo proteins into the vesicles (Barlowe, 2020; Barlowe and Miller, 2013; Kurokawa and Nakano, 2019; Phuyal and Farhan, 2021). For COPII vesicle formation, the small GTPase Sar1 is initially recruited to ER membrane by Sec12. Sec12 is an ER-resident protein that acts as a guanine nucleotide exchange factor (GEF) to catalyze the exchange of bound GDP to GTP on Sar1 (Barlowe and Schekman, 1993; Nakano and Muramatsu, 1989). The inner coat subunit Sec23–Sec24 complex is targeted to the ER membrane, and then recruits the outer coat Sec13–Sec31 complex (Matsuoka et al., 1998). In this process, the Sec23 subunit initially binds to membrane-bound Sar1 and later to Sec31 (Bi et al., 2002, 2007), whereas Sec24 captures the cargo molecules (Miller et al., 2002, 2003). These reactions are repeated, and polymerization of the Sec13–Sec31 complex finally occurs to drive vesicle formation (Stagg et al., 2006; Tabata et al., 2009). To ensure the formation of the cargo-loaded vesicles, Sec23 serves as a GTPase-activating protein (GAP) and activates Sar1 GTPase to selectively dissociate the cargo-uncaptured Sar1–Sec23–Sec24 complex from membranes (Sato and Nakano, 2005; Yoshihisa et al., 1993). Additionally, Sec31 can stimulate Sec23 GAP activity, which is thought to dissociate Sar1 from forming vesicles (Antonny et al., 2001; Iwasaki et al., 2017). A peripheral ER membrane protein, Sec16, is proposed to counter this reaction by inhibiting Sec31-simulated Sec23 GAP, which stabilizes the coat complex assembled on membranes to facilitate vesicle formation (Kung et al., 2012; Supek et al., 2002; Yorimitsu and Sato, 2012). Sec16 has been shown to have binding sites for multiple COPII proteins, and to localize to ERES along with them (Espenshade et al., 1995; Shaywitz et al., 1997). Given that these proteins are conserved from lower eukaryotes to mammals, the basic mechanisms for transport carrier formation at the ERES are shared across species.

The ER network consists of tubular and sheet structures (Shibata et al., 2009). ERES have been found to be specifically generated in the high-curvature regions of the ER, such as the tubules and edges of the sheets (Hammond and Glick, 2000; Okamoto et al., 2012). Compared to our understanding of vesicle formation reactions, however, the mechanisms for ERES definition and formation are poorly understood. We and others have previously shown that depletion or inactivation of Sec16 disrupts ERES, suggesting that Sec16 plays an important role in ERES formation as well as GTPase regulation (Connerly et al., 2005; Hughes et al., 2009; Ivan et al., 2008; Shindiapina and Barlowe, 2010; Sprangers and Rabouille, 2015; Yorimitsu and Sato, 2012). Thus, elucidating how Sec16 assembles on the ER membrane is important to address a key issue in ERES formation. Given that Sec12 acts as an initiator of COPII assembly at the most upstream reaction, inactivation of the temperature-sensitive Sec12 mutant at non-permissive temperatures perturbs ERES localization of COPII coats in *S. cerevisiae*. However, the ERES localization of Sec16 was not altered under these conditions (Okamoto et al., 2012; Shindiapina and Barlowe, 2010). In mammalian cells depleted of Sec12, Sec16 is also observed to be properly localized to the ERES (Saito et al., 2014). These results suggest that Sec16 localization is independent of Sec12 and the subsequent COPII assembly. In contrast, in *Pichia pastoris*, dissociation of COPII protein from ERES simultaneously dispersed Sec16 from ERES into the cytosol, suggesting that COPII assembly supports Sec16 localization to ERES (Bharucha et al., 2013). In mammalian and *Drosophila* cells, however, Sec16 remains at the ERES after depletion of Sec23 (Ivan et al., 2008; Maeda et al., 2017). These observations imply that the mechanisms of ERES formation and ERES localization of Sec16 vary to some extent among species. In fact, in mammals and *P. pastoris*, Sec12 localizes to the ERES, whereas in *S. cerevisiae*, Sec12 is localized to the general ER but not to the ERES (Okamoto et al., 2012; Saito et al., 2014; Soderholm et al., 2004). Additionally, in mammals, a metazoan-specific ER membrane protein, TANGO1, which was originally identified to function in pro-collagen export from the ER, has been suggested to function in ERES formation along with Sec16 (Maeda et al., 2017).

In *S. cerevisiae*, a Sec12 homolog, Sed4 was first isolated as a multicopy suppressor of depletion of the HDEL receptor Erd2 and later characterized as a component involved in ER export (Gimeno et al., 1995; Hardwick et al., 1992). Sed4 has now been found in some genomes from *Saccharomyces* and *Candida* species (Schlacht and Dacks, 2015). In a previous study, HA-tagged Sed4 was overexpressed from a multicopy plasmid and observed to localize throughout the ER using immunofluorescence microscopy (Gimeno et al., 1995). There is a 45% identity in the amino acid sequence of the cytosolic domain between Sed4 and Sec12. The cytosolic domain of Sed4 was also predicted to be structurally similar to that of Sec12 (Schlacht and Dacks, 2015). The crystal structure of the cytosolic domain of Sec12 was recently resolved and revealed a seven-bladed β-propeller fold. In these studies, a K^+^-ion-binding K-loop was found, which plays a critical role in GEF activity (McMahon et al., 2012). In contrast, the luminal domain of Sed4 shares no sequence similarity with Sec12. In earlier studies, Sed4 and Sec12 have been shown to undergo glycosylation in the luminal domain (Gimeno et al., 1995; Nakano et al., 1988). A glycoproteome analysis recently identified 55 O-mannosylation sites in Sed4 as well as 15 O-mannosylation and one N-glycosylation sites in Sec12 (Neubert et al., 2016). Recent studies have demonstrated the importance of post-translational modifications in the regulation of COPII proteins, such as phosphorylation and O-glycosylation in the cytosol (Bisnett et al., 2021). However, the functions of glycosylation of Sed4 and Sec12 are unknown.

Several lines of evidence indicate that Sed4 and Sec12 have different functions. A biochemical study has shown that, unlike Sec12, Sed4 can activate Sar1 GTPase, but has no GEF activity (Kodera et al., 2011). Genetic investigations have also indicated that Sed4 and Sec12 are functionally not exchangeable. The temperature-sensitive *sec16-2* mutant was synthetically defective in cell growth compared to the temperature-sensitive *sec12-4* mutant and *sed4*Δ mutant, but only Sed4 was shown to function as a multicopy suppressor of *sec16-2* (Gimeno et al., 1995). Moreover, by pulldown and yeast two-hybrid assays, the C-terminal fragment of Sec16 was observed to bind to the cytosolic domain of Sed4, but not to that of Sec12. Although the molecular mechanism has not yet been determined, the model proposes that Sec16 and Sed4 function together in the early steps of the vesicle formation reaction, in which Sed4 might promote Sec16 assembly on the ER membranes (Gimeno et al., 1995).

Here, we present evidence that Sec16 localizes to the ERES in a manner that is dependent on Sed4. We determined the Sed4- and Sec23-binding sites in the C-terminal region of Sec16. In addition, the cytosolic β-propeller blades numbers 1–3 of Sed4 were identified to bind to Sec16. The interaction with Sed4 is necessary for proper localization of Sec16 to the ERES. We also show that Sed4 is concentrated at the ERES, which requires interaction with Sec16 and the action of the luminal domain, which preferentially distributes Sed4 to high-curvature areas of the ER membrane. We further found that Sed4 interacts with itself, which involves both the cytosolic and luminal domains. O-mannosylations in the luminal domain are also required for this Sed4 self-interaction. These results provide insights into the mechanism of the interplay between Sec16 and Sed4 for their function at ERES.

## RESULTS

### Sed4 is required for Sec16 localization to ERES

**Fig. 1.**
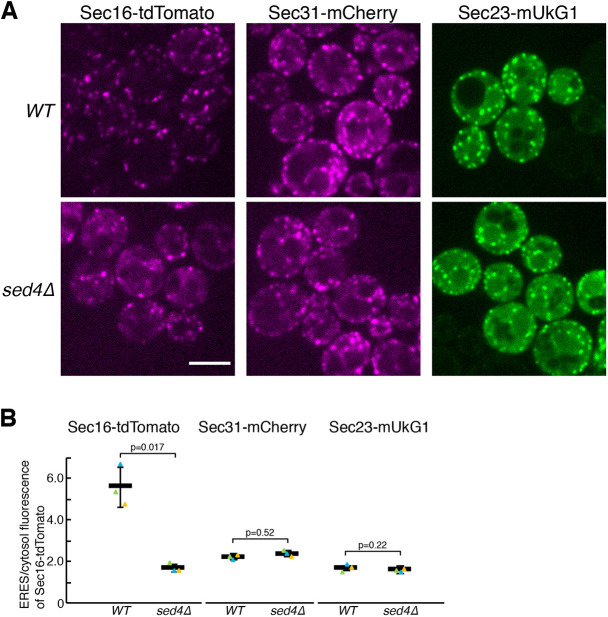
**Sed4 is required for localization of Sec16 to ERES.** (A) Wild-type and *sed4*Δ cells expressing Sec16–tdTomato, Sec31–mCherry or Sec23–mUkG1 were grown to a mid-log phase and observed by fluorescence microscopy. Scale bar: 4 μm. (B) Quantification of the relative intensity of Sec16–tdTomato localizing at ERES. Sec16–tdTomato, Sec31–mCherry or Sec23–mUkG1 were visualized in wild-type cells and *sed4*Δ cells as described in A, and the fluorescence intensities of each protein in ERES and in cytosol were measured with ImageJ (*n*=3 experiments, at least 40 cells observed). Error bar represents standard deviation. *P*-value was calculated with an unpaired two-tailed *t*-test.

We first aimed to determine whether Sed4 plays a role in ERES localization of Sec16 in *S. cerevisiae*. For this purpose, we visualized Sec16–tdTomato in *sed4*Δ cells by fluorescence microscopy. As a control, Sec16–tdTomato was observed to localize to punctate ERES in wild-type cells. We also observed the distribution of the COPII subunits Sec31–mCherry and Sec23–mUkG1 to the ERES in wild-type cells. Sec31–mCherry and Sec23–mUkG1 both yielded cytosolic staining, whereas Sec16–tdTomato displayed little to no signal in the cytosol (Fig. 1A,B). In contrast, in *sed4*Δ cells, Sec16–tdTomato was detected at the ERES but also exhibited a substantial diffuse cytosolic signal, compared to that in wild-type cells. The distribution patterns of Sec31–mCherry and Sec23–mUKG1 were substantially unchanged in *sed4*Δ cells. These results indicate that Sed4 is involved in the localization of Sec16 to the ERES.

### Sed4 is concentrated at ERES and interacts with Sec16 independently of the HDEL sequence at the C-terminal end

**Fig. 2.**
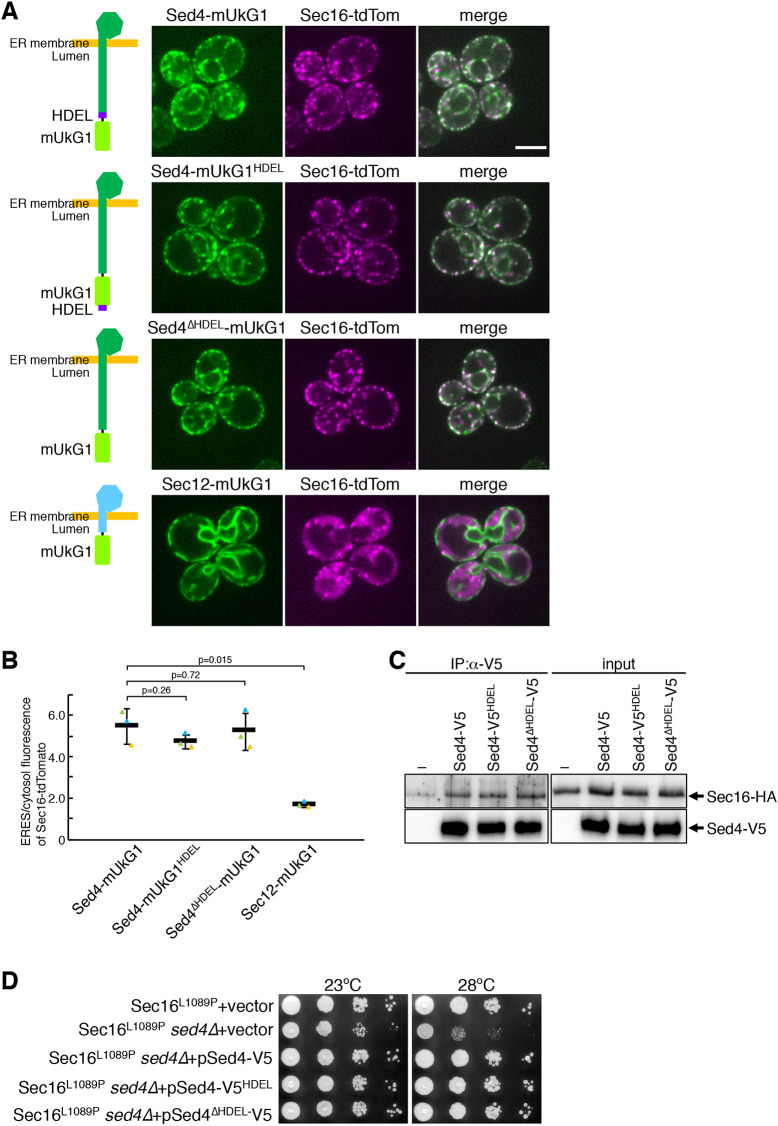
**The HDEL sequence is dispensable for Sed4 to mediate concentration of Sec16 at ERES and for its interaction with Sec16.** (A) *sed4*Δ *sec16*Δ cells expressing Sec16–tdTomato with Sed4–mUkG1, Sed4–mUkG1^HDEL^, Sed4^ΔHDEL^–mUkG1 or Sec12–mUkG1 were grown to mid-log phase and observed by fluorescence microscopy. The mUKG1-fused Sed4 or Sec12 constructs are depicted in the left panel of each image. The mUkG1 and HDEL sequences are represented in light green and purple, respectively (see also Fig. 3A). Scale bar: 4 μm. (B) Quantification of the relative intensity of Sec16–tdTomato localizing at ERES. Sec16–tdTomato was visualized in *sed4*Δ *sec16*Δ cells co-expressing Sed4–mUkG1, Sed4–mUkG1^HDEL^, Sed4^ΔHDEL^–mUkG1 or Sec12–mUkG1 as described in A, and the fluorescence intensities of Sec16–tdTomato in ERES and cytosol were measured with ImageJ (*n*=3 experiments, at least 40 cells observed). Error bar represents standard deviation. *P-*value was calculated with an unpaired two-tailed *t*-test. (C) *sed4*Δ *sec16*Δ cells expressing Sec16–HA with a control vector, Sed4–V5, Sed4–V5^HDEL^, Sed4^ΔHDEL^–V5 or Sec12–V5 were grown and collected at mid-log phase. Each V5-tagged Sed4 was constructed analogously to mUkG1-fused Sed4 used in A. Octylglucoside-solubilized cell extracts were subjected to immunoprecipitation with anti-V5 antibody, and precipitated proteins were analyzed by immunoblotting with anti-HA and anti-V5 antibodies. Input, 0.1%. (D) Serial dilution of *sec16*Δ cells expressing Sec16^L1089P^ transformed with a control vector and *sed4*Δ *sec16*Δ cells expressing Sec16^L1089P^ transformed with a control vector or a plasmid harboring Sed4–V5, Sed4–V5^HDEL^ or Sed4^ΔHDEL^–V5 were incubated on plates at 23°C and 28°C for 2 days and imaged. Images shown in C and D are representative of three repeats.

A previous study has shown that Sed4 is present in the ER, but it has not been determined whether Sed4 localizes to the ERES (Gimeno et al., 1995). To address this issue, we observed *sed4*Δ cells expressing Sed4–mUkG1 from the endogenous promoter on a low-copy plasmid using fluorescence microscopy. Sec16–tdTomato was visualized together in these cells and found to properly localize to ERES (Fig. 2A,B), comparable to that observed in wild-type cells (Fig. 1), indicating that Sed4–mUkG1 mediates Sec16 localization to the ERES to the same level as untagged endogenous Sed4. Sed4–mUKG1 was distributed to the general ER and simultaneously displayed punctate structures in the ER that colocalized with Sec16–tdTomato (Fig. 2A; Fig. S1). These results indicate that Sed4–mUKG1 was concentrated at the ERES. However, when co-expressed with Sec12–mUKG1 in *sed4*Δ cells, as observed in *sed4*Δ cells in Fig. 1, Sec16–tdTomato showed ERES localization and a significant cytosolic distribution. In these cells, Sec12–mUKG1 was detected throughout the ER without accumulation at the ERES, as reported previously (Okamoto et al., 2012). This is consistent with previous observations that Sed4 and Sec12 play different roles and are unexchangeable.

**Fig. 3.**
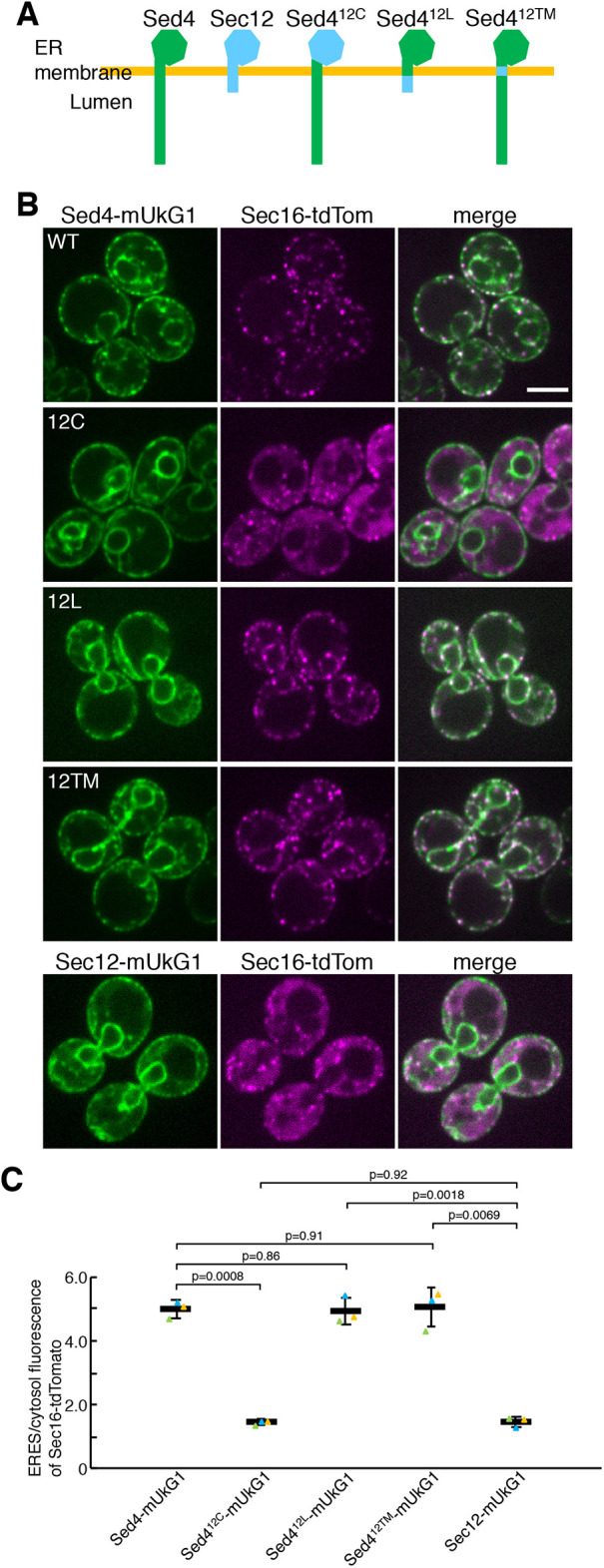
**The cytosolic domain of Sed4 is essential for ERES localization of Sed4 and Sec16, and the luminal domain is necessary for ERES localization of Sed4.** (A) Schematic diagram of Sed4–Sec12 chimera constructs, in which Sed4- and Sec12-derived portions are represented in green and light blue, respectively. (B) *sed4*Δ *sec16*Δ cells expressing Sec16–tdTomato with Sed4–mUkG1 (wild type; WT), Sed4^12C^–mUkG1 (12C), Sed4^12L^–mUkG1 (12L), Sed4^12TM^–mUkG1 (12TM) or Sec12–mUkG1 were grown to a mid-log phase and observed by fluorescence microscopy. Scale bar: 4 μm. (C) Quantification of the relative intensity of Sec16–tdtomato localizing at ERES. Sec16–tdTomato was visualized in *sed4*Δ *sec16*Δ cells co-expressing Sed4–mUkG1, Sed4^12C^–mUkG1, Sed4^12L^–mUkG1, Sed4^12TM^–mUkG1 or Sec12–mUkG1 as described in A, and the fluorescence intensities of Sec16-tdTomato in ERES and cytosol were measured with ImageJ (*n*=3 experiments, at least 40 cells observed). Error bar represents standard deviation. *P*-value was calculated with an unpaired two-tailed *t*-test.

Sed4 has an HDEL sequence at its C-terminal end. Although the HDEL sequence usually acts as an ER retention signal (Pelham et al., 1988), its role in Sed4 function is unclear. The Sed4–mUKG1 used in Fig. 2 has an mUKG1 fusion immediately after the HDEL sequence, so the fusion could potentially affect the HDEL functionality, causing an abnormal concentration of Sed4 at the ERES. To test this possibility, we created additional constructs, Sed4–mUKG1^HDEL^ and Sed4^ΔHDEL^–mUKG1. Sed4–mUKG1^HDEL^ has an mUKG1 fusion just preceding the HDEL sequence, which can be expected to be exposed and functional, and Sed4^ΔHDEL^–mUKG1 lacks the HDEL sequence. These constructs were expressed with Sec16–tdTomato in *sed4*Δ cells and their distribution was compared with that of Sed4–mUKG1 (Fig. 2A,B). As observed with Sed4–mUKG1, both constructs were found to enable Sec16–tdTomato to properly localize to ERES and to be concentrated at ERES with general ER staining. These results suggest that ERES localization of Sed4 with Sec16 is independent of the HDEL sequence.

We continued to test the requirement of the HDEL sequence for the Sed4 function. In a previous study, an interaction between Sed4 and Sec16 was observed using fragment constructs (Gimeno et al., 1995). Thus, we examined the interaction between full-length constructs of Sed4 and Sec16. For this analysis, we tagged Sed4 with the V5 epitope analogously to the mUkG1 constructs, and created Sed4–V5, Sed4–V5^HDEL^ and Sed4^ΔHDEL^–V5. Octylglucoside-solubilized extracts were prepared from cells expressing these constructs with Sec16–HA and co-immunoprecipitation assays were performed using anti-V5 antibody (Fig. 2C). Sec16–HA was found to be co-precipitated with each Sed4 construct at a similar level.

Next, we performed a complementation assay using Sec16^L1089P^ *sed4*Δ cells, which are *sec16*Δ *sed4*Δ cells expressing a temperature-sensitive Sec16^L1089P^ mutant. As reported previously (Gimeno et al., 1995), with a control vector, Sec16^L1089P^ *sed4*Δ cells displayed synthetic growth defects at permissive (23°C) and semi-permissive (28°C) temperatures, compared with Sec16^L1089P^ cells (*sec16*Δ cells expressing Sec16^L1089P^). All Sec16^L1089P^ *sed4*Δ cells expressing Sed4–V5, Sed4–V5^HDEL^ and Sed4^ΔHDEL^–V5 no longer displayed growth defects at 23°C and 28°C and grew to the same level as Sec16^L1089P^ cells. These results indicate that the HDEL sequence is not required for the Sed4 function accomplished with Sec16.

### The cytosolic domain of Sed4 is essential for ERES localization of Sed4 and Sec16

To characterize which portion of Sed4 is required for the concentration of Sed4 and Sec16 at the ERES, we created chimeric constructs in which the domain of Sed4 was replaced with the corresponding domain of Sec12 (Fig. 3A). These constructs were fused with mUkG1 and visualized with Sec16–tdTomato in *sed4*Δ cells using fluorescence microscopy (Fig. 3B,C). With Sed4^12L^–mUkG1 and Sed4^12TM^–mUkG1, which have replaced luminal and transmembrane domains, respectively, Sec16–tdTomato is localized to the ERES normally, as observed with wild-type Sed4–mUkG1. Sed4^12TM^–mUkG1 was distributed at the ERES, which is comparable to Sed4–mUkG1, whereas Sed4^12L^–mUkG1 was not properly concentrated at the ERES. In contrast, with Sed4^12C^–mUkG1, which has a Sec12-derived cytosolic domain, Sec16–tdTomato displayed significant cytosolic staining together with ERES localization, as seen with Sec12–mUkG1. Sed4^12C^–mUkG1 localized to the entire ER but not to the ERES. To confirm the distribution pattern of these chimera proteins, the mScarlet-fused constructs were observed with the ER marker Sec71–EGFP by focusing on the periphery of the cells (Fig. S2). As reported previously (Voeltz et al., 2006), the peripheral ER network was marked by Sec71–EGFP. Sed4^12C^–mScarlet and Sed4^12L^–mScarlet were found to show some foci but to be mainly distributed throughout the ER network, similar to Sec12–mScarlet. Sed4^12TM^–mScarlet, as well as Sed4–mScarlet, was predominantly concentrated at the ERES, as observed with Sed4–mUkG1 in Fig. S1B. These observations suggest that the cytosolic domain of Sed4 acts as the main determinant of ERES localization of Sed4 and Sec16, and the luminal domain also plays a role in the concentration of Sed4 at the ERES.

### Identification of the interaction sites between Sec16 and Sed4

**Fig. 4.**
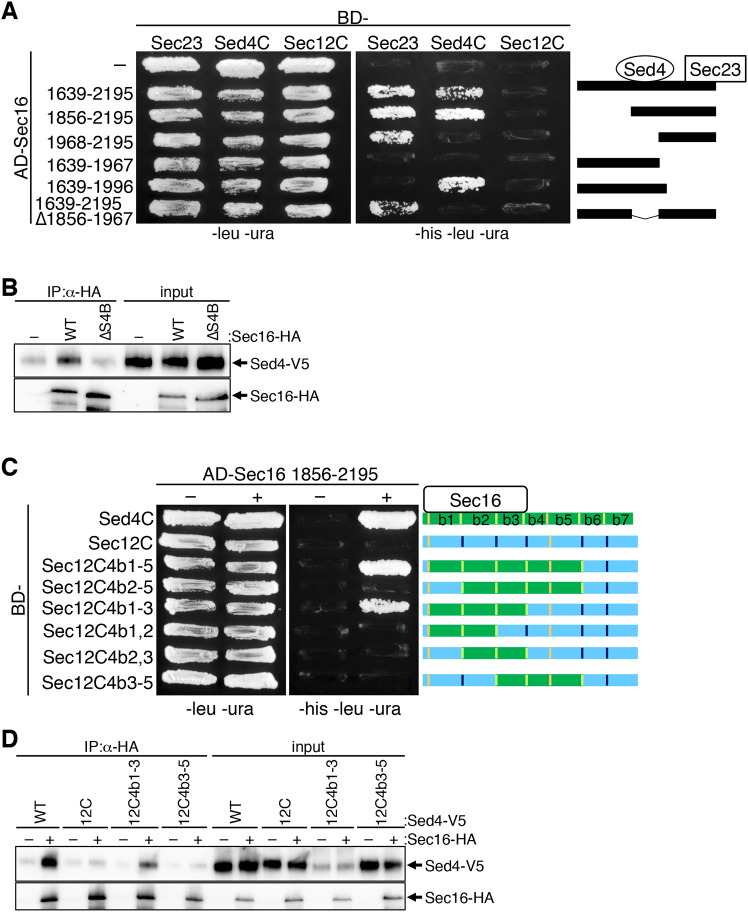
**Interaction between Sec16 and Sed4.** (A) Yeast two-hybrid analysis was performed to explore the binding sites for Sed4 and Sec23 in Sec16. A yeast two-hybrid assay strain was transformed with a control vector, or plasmids containing the activation domain (AD)-fused Sec16 fragment indicated along with plasmids containing the binding domain (BD)-fused Sec23, Sed4C (the N-terminal cytosolic domain of Sed4) or Sec12C (the N-terminal cytosolic domain of Sec12), and incubated on plates lacking leucine and uracil (−leu −ura), or histidine, leucine and uracil (−his −leu −ura) at 30°C for 4 days. Numbers of each fragment represent amino acid residues of Sec16. The fragments of the Sec16 C-terminal region tested here are depicted in the right panel. (B) *sed4*Δ *sec16*Δ cells expressing Sed4–V5 with or without Sec16–HA (wild type; WT) or Sec16^ΔS4B^–HA (ΔS4B) were grown and collected at mid-log phase. Immunoprecipitation was performed with anti-HA antibody, and precipitated proteins were analyzed by immunoblotting with anti-HA and anti-V5 antibodies, as described in Fig. 2C. (C) Yeast two-hybrid analysis was performed to explore the binding sites for Sec16 in Sed4. The yeast two-hybrid assay strain was transformed with a control vector or plasmids containing the activation domain (AD)-fused 1856–2195 fragment of Sec16 along with plasmids containing the binding domain (BD)-fused Sed4C, Sec12C, or chimera constructs indicated and incubated on −leu −ura or −his −leu −ura plates at 30°C for 4 days. Chimera constructs are depicted in the right panel, in which the β-propeller blades of Sec12C were replaced by the corresponding blades of Sed4C. (D) *sed4*Δ *sec16*Δ cells expressing Sed4–V5 (WT), Sed4^12C^–V5 (12C), Sed4^12C4b1-3^–V5 (12C4b1-3) or Sed4^12C4b3-5^–V5 (12C4b3-5) with or without Sec16–HA were grown and collected at mid-log phase. After immunoprecipitation, precipitated proteins were analyzed, as described in B. Images shown in this figure are representative of three repeats.

The cytosolic domain of Sed4 has been reported to interact with the C-terminal region of Sec16 (Gimeno et al., 1995). Therefore, we speculated that the interaction between Sec16 and Sed4 participates in their localization to the ERES. To prove this hypothesis, we first sought to determine the interaction sites of the two proteins. For this purpose, we began with the C-terminal fragment consisting of amino acid residues 1639–2195 of Sec16, which corresponds to the fragment used in a previous study (Gimeno et al., 1995). Using yeast two-hybrid analysis, this fragment was found to bind to the N-terminal cytosolic domain of Sed4 (Sed4C) and Sec23 (Fig. 4A), but not to the N-terminal cytosolic domain of Sec12 (Sec12C), as reported previously (Espenshade et al., 1995; Gimeno et al., 1995). By dissecting this fragment, we found that the fragment with residues 1856–2195 binds to Sed4C and Sec23, and the fragment with residues 1968–2195 interacts with Sec23 but not with Sed4C. In contrast, the fragment with residues 1639–1996 was observed to bind to Sed4C but not to Sec23. The fragment with residues 1639–1967 bound neither to Sec23 nor Sed4C. These results imply that amino acid residues 1856–1967 contain a Sed4-binding site. This was confirmed by the observation that the 1639–2195 Δ1856–1967 fragment, which consists of residues 1639–2195 but lacking residues 1856–1967, continues to bind to Sec23 but fails to interact with Sed4C. None of the constructs of Sec16 examined exhibited an interaction with Sec12C. These results indicate that Sec16 has two distinct binding sites for Sed4 and Sec23 at the C-terminus.

We then examined the Sed4-binding site in the full-length Sec16. As described in Fig. 2C, we prepared lysates of cells expressing Sed4–V5 with wild-type Sec16–HA or the Sec16^ΔS4B^–HA mutant, which lacks the Sed4-binding site at amino acid residues 1856–1967 and performed co-immunoprecipitation assays using anti-HA antibody (Fig. 4B). Compared with Sec16–HA, Sed4–V5 was co-precipitated with Sec16^ΔS4B^–HA only at the background level, equivalent to that with a control vector. Therefore, the region of Sec16 identified by the yeast two-hybrid assay is the Sed4-binding site in the full-length Sec16.

Next, we investigated the binding site for Sec16 in Sed4. In the yeast two-hybrid assay, the Sec16 fragment consisting of residues 1856–2195 was found to interact with Sed4C but not with Sec12C (Fig. 4A). Thus, we replaced the β-propeller blade domains of Sec12C with the corresponding domains of Sed4C and sought a Sec12C chimera construct that is able to bind to Sec16. Sec12C4b1-5, in which blade numbers 1–5 (#1–5) of Sec12 were replaced with the corresponding blades of Sed4, successfully interacted with Sec16, indicating that blades #1–5 of Sed4 include a Sec16-binding site (Fig. 4C). To narrow down the binding sites of these blades, we created additional Sec12C chimera constructs. Among these constructs, only Sec12C4b1-3, which contains replaced blades #1–3 of Sed4, was found to bind to Sec16. Because Sec12C4b1-2 and Sec12C4b2-3 exhibited no interaction, blades #1–3 were suggested to be the minimal requirement for the interaction. To verify these results, we created Sed4^12C4b1-3^–V5 and Sed4^12C4b3-5^–V5, in which the cytosolic domain of Sed4–V5 was replaced by Sec12C4b1-3 and Sec12C4b3-5, respectively. Co-immunoprecipitation assays revealed that Sec16–HA interacted with Sed4^12C4b1-3^–V5 to the same level as wild-type Sed4-V5, although, for unknown reasons, in the input lysate, Sed4^12C4b1-3^–V5 was detected at a lower level than Sed4–V5 (Fig. 4D). Sed4^12C^–V5 and Sed4^12C4b3-5^–V5 were detected at the same level as Sed4–V5 in the lysates, but neither showed an interaction with Sec16–HA. These findings are consistent with the results of the yeast two-hybrid assay. Collectively, we conclude that β-propeller blades #1–3 of Sed4 mediate the interaction with Sec16.

### The interaction between Sec16 and Sed4 is essential for their localization to ERES

**Fig. 5.**
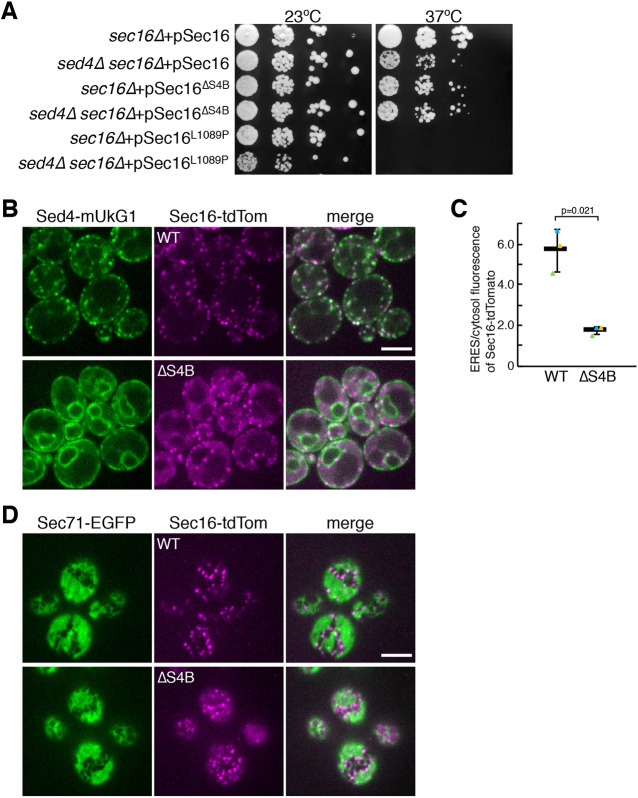
**Interaction with Sed4 is required for Sec16 to mediate ERES localization of Sec16 and Sed4.** (A) Serial dilution of *sec16*Δ cells and *sed4*Δ *sec16*Δ cells transformed with a plasmid harboring wild-type Sec16, Sec16^ΔS4B^ or Sec16^L1089P^ were incubated on plates at 23°C and 37°C for 3 days. (B) *sed4*Δ *sec16*Δ cells expressing Sed4–mUkG1 with Sec16–tdTomato (wild type; WT) or Sec16^ΔS4B^-tdTomato (ΔS4B) were grown to a mid-log phase and observed by fluorescence microscopy. Scale bar: 4 μm. (C) Quantification of the relative intensity of Sec16–tdtomato and Sec16^ΔS4B^–tdTomato localizing at ERES. Sec16–tdTomato and Sec16^ΔS4B^–tdTomato were visualized in *sed4*Δ *sec16*Δ cells co-expressing Sed4–mUkG1 as described in B, and the fluorescence intensities of Sec16–tdTomato or Sec16^ΔS4B^–tdTomato in ERES and cytosol were measured with ImageJ (*n*=3 experiments, at least 40 cells observed). Error bar represents standard deviation. *P*-value was calculated with an unpaired two-tailed *t*-test. (D) *rtn1*Δ *rtn2*Δ *yop1*Δ *sec16*Δ cells expressing Sec71–EGFP with Sec16–tdTomato (WT) or Sec16^ΔS4B^–tdTomato (ΔS4B) were grown to a mid-log phase and observed by fluorescence microscopy. The ER and ERES were visualized by focusing on the periphery of the cell. Scale bar: 4 μm. Images in A and D are representative of three repeats.

We then investigated whether the interaction between Sec16 and Sed4 links their functions. To test this, we expressed Sec16, Sec16^ΔS4B^ and the temperature-sensitive mutant Sec16^L1089P^ in *sec16*Δ or *sed4*Δ *sec16*Δ cells, and performed a growth assay at permissive (23°C) and non-permissive (37°C) temperatures (Fig. 5A). At 37°C, *sed4*Δ *sec16*Δ cells expressing Sec16 grew slower than *sec16*Δ cells expressing Sec16, whereas *sed4*Δ *sec16*Δ cells and *sec16*Δ cells expressing Sec16^L1089P^ were not viable. At 37°C, a temperature-sensitive growth defect was observed in both *sed4*Δ *sec16*Δ cells and *sec16*Δ cells expressing Sec16^ΔS4B^. We also examined the distribution of Sed4–mUkG1 with Sec16^ΔS4B^–tdTomato (Fig. 5B,C). In these cells, Sed4–mUkG1 was observed to localize to the general ER but was not concentrated at the ERES, and Sec16^ΔS4B^–tdTomato displayed diffuse cytosolic staining with some ERES localization, similar to what is seen for Sec16–tdTomato in *sed4*Δ cells (Fig. 1A). These findings suggest that the lack of Sed4 binding compromises Sec16 function and causes a failure to concentrate Sed4 at the ERES.

We examined whether ERES were properly formed by Sec16^ΔS4B^ in the high-curvature regions of the ER. Although *S. cerevisiae* is rich in tubular ER, depletion of the ER-shaping proteins Rtn1, Rtn2 and Yop1, dramatically changes the ER structure, in which the tubules are reduced, and the sheets are expanded (Voeltz et al., 2006). Sec16–tdTomato and Sec16^ΔS4B^–tdTomato were coexpressed with Sec71–EGFP in *rtn1*Δ *rtn2*Δ *yop1*Δ cells, and peripheral ER was observed by focusing on the periphery of the cells, as described previously (Okamoto et al., 2012). Sec71–EGFP was present throughout the ER, including in tubules and sheets. We found that ERES visualized by Sec16^ΔS4B^–tdTomato were present in the tubules and the edges of the sheets of the peripheral ER, as visualized by Sec16–tdTomato (Fig. 5D), suggesting that loss of interaction with Sed4 does not prevent Sec16 from limiting ERES formation to the regions of the high-curvature membrane of the ER.

**Fig. 6.**
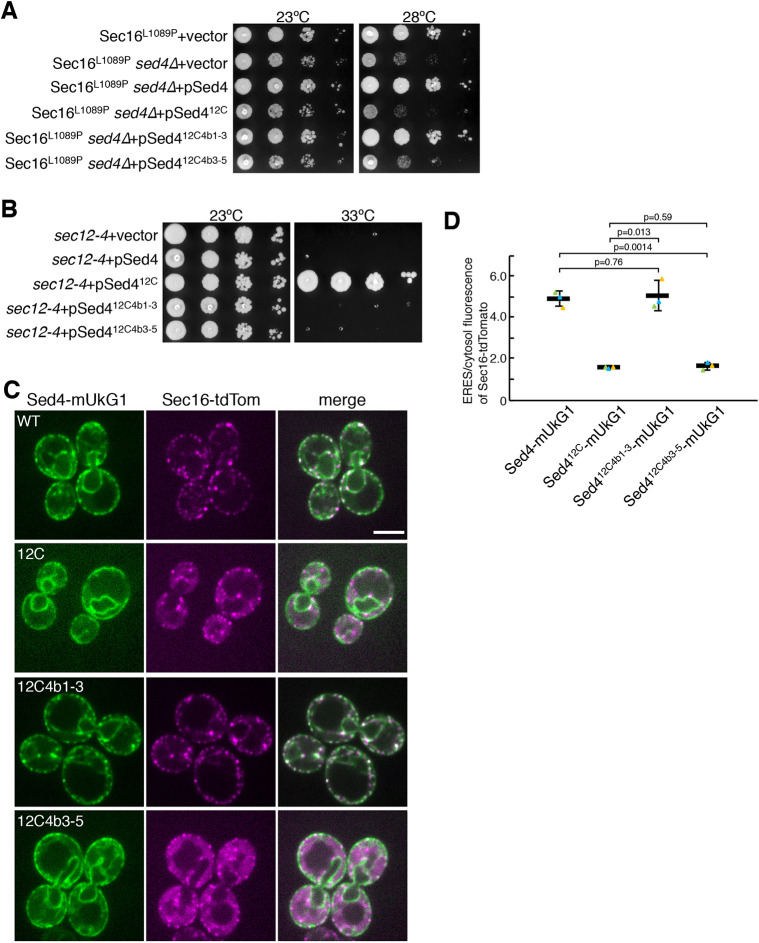
**Interaction with Sec16 is required for Sed4 to mediate ERES localization of Sed4 and Sec16.** (A) Serial dilution of *sec16*Δ cells expressing Sec16^L1089P^ transformed with a control vector, and *sed4*Δ *sec16*Δ cells expressing Sec16^L1089P^ transformed with a control vector or a plasmid harboring Sed4, Sed4^12C^, Sed4^12C4b1-3^ or Sed4^12C4b3-5^ were incubated on plates at 23°C and 28°C for 2 days. (B) Serial dilution of *sec12-4* cells transformed with a control vector or a plasmid harboring Sed4, Sed4^12C^, Sed4^12C4b1-3^ or Sed4^12C4b3-5^ were incubated on plates at 23°C for 3 days and at 33°C for 2 days. (C) *sed4*Δ *sec16*Δ cells expressing Sec16–tdTomato with Sed4–mUkG1 (wild type; WT), Sed4^12C^–mUkG1 (12C), Sed4^12C4b1-3^–mUkG1 (12C4b1-3), or Sed4^12C4b3-5^–mUkG1 (12C4b3-5) were grown to a mid-log phase and observed by fluorescence microscopy. Scale bar: 4 μm. (D) Quantification of the relative intensity of Sec16–tdtomato localizing at ERES. Sec16–tdTomato were visualized in *sed4*Δ *sec16*Δ cells co-expressing Sed4–mUkG1 (WT), Sed4^12C^–mUkG1 (12C), Sed4^12C4b1-3^–mUkG1 (12C4b1-3), or Sed4^12C4b3-5^–mUkG1 (12C4b3-5), as described in B, and the fluorescence intensities of Sec16–tdTomato in ERES and cytosol were measured with ImageJ (*n*=3 experiments, at least 40 cells observed). Error bar represents standard deviation. *P*-value was calculated with an unpaired two-tailed *t*-test. Images in A and B are representative of three repeats.

Next, we tested the function of the Sed4–Sec12 chimeric constructs in *sed4*Δ cells. As shown in Fig. 2D, each construct was expressed in Sec16^L1089P^ *sed4*Δ cells, and growth was assessed at 23°C and 28°C (Fig. 6A). Under both temperature conditions, Sed4^12C4b1-3^ supported the growth of Sec16^L1089P^ *sed4*Δ cells to the same level as Sed4. On the other hand, Sec16^L1089P^ *sed4*Δ cells expressing Sed4^12C^ and Sed4^12C4b3-5^, which are unable to bind Sec16, still exhibited growth defects at 23°C and 28°C, similar to in the cells carrying a control vector. These results suggest that Sed4^12C4b1-3^ can act as Sed4. The growth test was performed in temperature-sensitive *sec12-4* cells. As reported previously (Gimeno et al., 1995), *sec12-4* cells with a plasmid expressing Sed4 and a control vector were viable at 23°C but not at 33°C. Sed4^12C^ was able to rescue growth at 33°C as well as at 23°C, whereas neither Sed4^12C4b1-3^ nor Sed4^12C4b3-5^ conferred viability at 33°C (Fig. 6B), suggesting that Sed4^12C4b1-3^ and Sed4^12C4b3-5^ lose the ability to exert the essential function of Sec12. We examined the distribution of mUkG1-fused Sed4^12C4b1-3^ and Sed4^12C4b3-5^ with Sec16–tdTomato in *sed4*Δ cells. Sed4^12C4b1-3^–mUkG1 enabled Sec16–tdTomato to properly localize to the ERES to the level that Sed4–mUkG1 did, and to be concentrated there. Similar to Sed4^12C^–mUkG1, Sed4^12C4b3-5^–mUkG1 did not localize to the ERES and exhibited a diffuse cytosolic distribution of Sec16–tdTomato (Fig. 6C,D). Consequently, these findings indicate that the interaction between Sec16 and Sed4 is necessary for their function, including ERES localization.

### Preferential localization of Sed4 at high-curvature membrane

**Fig. 7.**
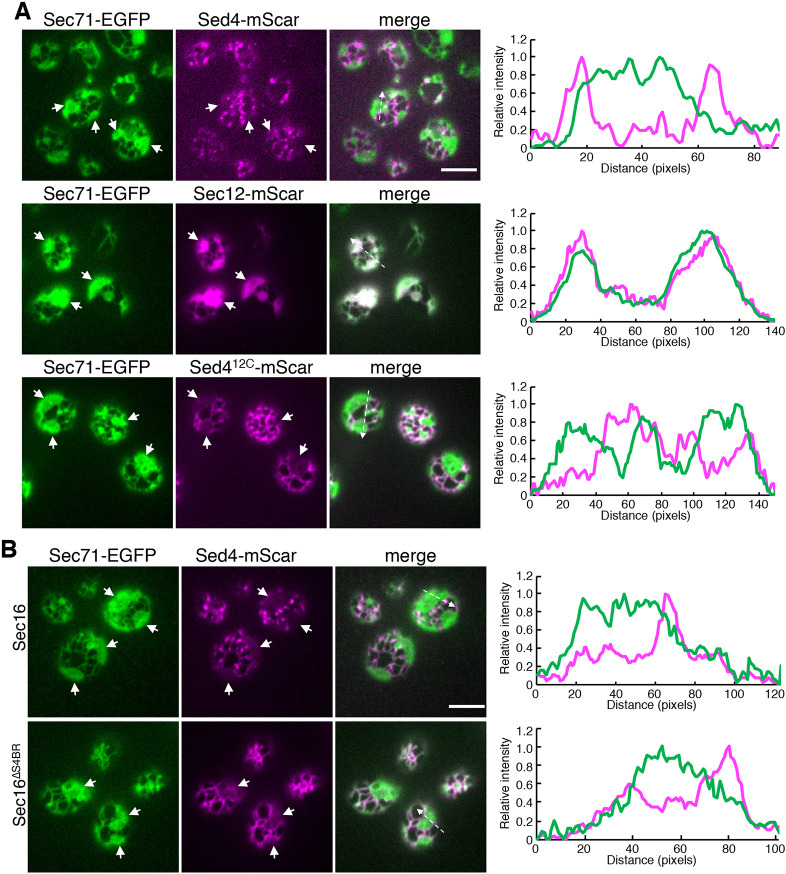
**Loss of interaction with Sec16 redistributes Sed4 from being preferentially located at ERES to being at the tubules and edges of the sheets of the ER.** (A) *rtn1*Δ *rtn2*Δ *yop1*Δ cells expressing Sec71–EGFP with Sed4–mScarlet, Sed4^12C^–mScarlet or Sec12–mScarlet were grown to a mid-log phase and observed by fluorescence microscopy. The ER and ERES were visualized by focusing on the periphery of the cell. (B) *rtn1*Δ *rtn2*Δ *yop1*Δ *sec16*Δ cells expressing Sec71–EGFP and Sed4–mScarlet with Sec16 or Sec16^ΔS4B^ were grown to a mid-log phase and observed by fluorescence microscopy. The ER and ERES were visualized by focusing on the periphery of the cell. White arrows indicate the sheet regions of the ER. Line-scan analysis was carried out at the area indicated by a white dashed arrow in the merged images, and profile plots of the normalized intensity are shown on the right. Scale bars: 4 μm. Images are representative of three repeats.

Sed4 was found to be distributed in two pools – one located at ERES and one located at general ER. To examine whether Sed4 localizes to the entire ER, including tubules and sheets, we visualized Sed4–mScaret with Sec71–EGFP in *rtn1*Δ *rtn2*Δ *yop1*Δ cells and observed the peripheral ER, as described in Fig. 5. Unlike Sec71–EGFP, Sed4–mScarlet localized to the ERES and to the tubules and edges of the sheets but exhibited very weak or no signal at the sheets (Fig. 7A; Fig. S3A). In contrast, as observed previously (Okamoto et al., 2012), Sec12–mScarlet overlapped with Sec71–EGFP and was uniformly distributed in the tubules and sheets. The distribution of Sed4^12C^–mUkG1 was similar to that of Sec12–mUkG1 (Fig. 3B). When observed in *rtn1*Δ *rtn2*Δ *yop1*Δ cells, however, Sed4^12C^–mScarlet displayed limited localization to the tubules and the edges of the sheets, but not to the sheets. Because Sed4^12C^ loses its interaction with Sec16, we also tested the distribution of Sed4–mScarlet in *rtn1*Δ *rtn2*Δ *yop1*Δ *sec16*Δ cells expressing Sec16 or Sec16^ΔS4B^ (Fig. 7B; Fig. S3B). With Sec16, Sed4–mScarlet was primarily detected at the ERES, located at the tubules and edges of the sheets. On the other hand, with Sec16^ΔS4B^, Sed4–mScarlet did not display punctate ERES structures, but remained exclusively present throughout the tubules and the edges of the sheets. These results indicate that upon loss of interaction with Sec16, Sed4 is not concentrated at the ERES but remains in the high-curvature areas of the ER.

Because Sec12 does not exhibit such a localization pattern, we speculated that the luminal domain of Sed4 mediates the limited distribution to high-curvature areas. To test this, we created sets of mutants of Sed4^12C^–mScarlet with a truncated luminal domain and visualized them in *rtn1*Δ *rtn2*Δ *yop1*Δ cells (Fig. S4A,B). Among the mutants, only Sed4^12CΔ942L^–mScarlet, which lacks 124 amino acids in the C-terminal region, was localized to the tubules and edges of the sheets, suggesting that the truncated region is indispensable for localization to the high-curvature areas. The remaining mutants were distributed throughout the ER. These results suggest that the limited localization of Sed4^12C^ to high-curvature areas is mediated by almost the entire region or multiple regions of the luminal domain, but not by one narrow region.

### Sed4 interacts with itself independently of ERES localization

**Fig. 8.**
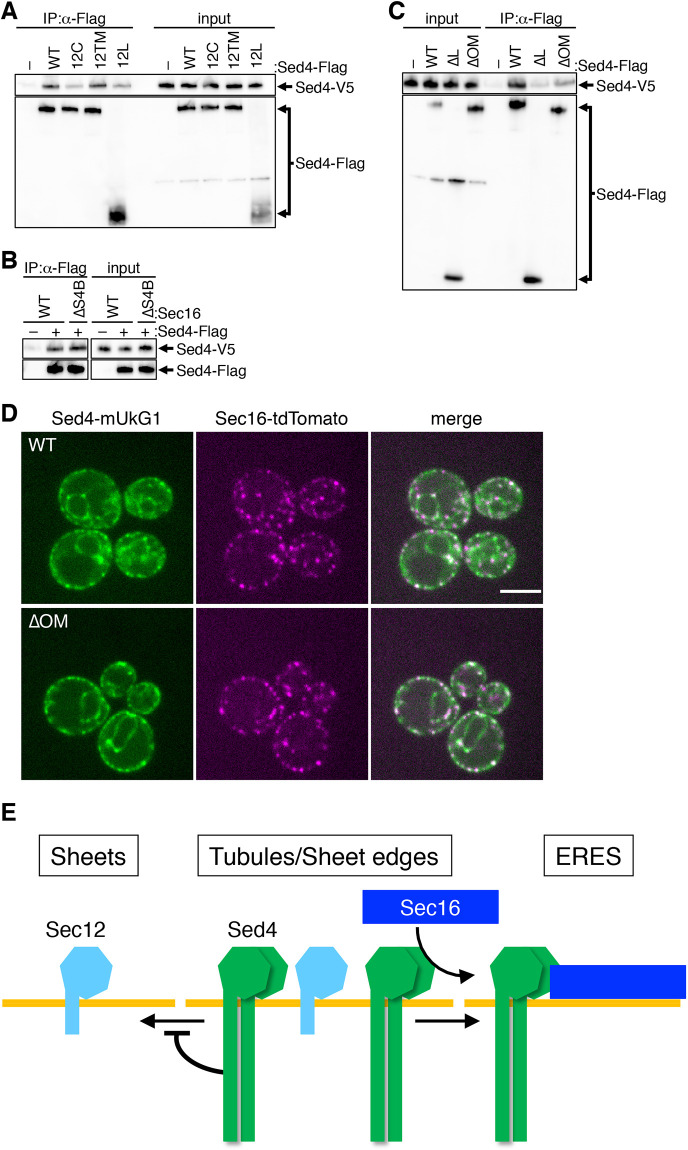
**Sed4 interacts with itself, which requires the cytosolic and luminal domains and O-mannosylations in the luminal domain.** (A) *sed4*Δ cells expressing Sed4–V5 with or without Sed4–Flag (wild type; WT), Sed4^12C^–Flag (12C), Sed4^12TM^–Flag (12TM) or Sed4^12L^–Flag (12L) were grown and collected at mid-log phase. Immunoprecipitation was performed with anti-Flag antibody, and precipitated proteins were analyzed by immunoblotting with anti-Flag and anti-V5 antibodies, as described in Fig. 2C. (B) *sed4*Δ *sec16*Δ cells expressing Sec16 (WT) and Sed4–V5 with or without Sed4–Flag, or *sed4*Δ *sec16*Δ cells expressing Sec16ΔS4B (ΔS4B) and Sed4–V5 with Sed4–Flag were grown and collected at mid-log phase. After immunoprecipitation, precipitated proteins were analyzed, as described in A. (C) *sed4*Δ cells expressing Sed4–V5 with or without Sed4–Flag (WT), Sed4^ΔL^–Flag (ΔL), or Sed4^ΔOM^–Flag (ΔOM) were grown and collected at mid-log phase. After immunoprecipitation, precipitated proteins were analyzed, as described in A. Input, 0.05%. (D) *sed4*Δ *sec16*Δ cells expressing Sec16–tdTomato with Sed4–mUkG1 (WT) or Sed4^ΔOM^–mUkG1 (ΔOM) were grown to a mid-log phase and observed by fluorescence microscopy. Scale bar: 4 μm. Images in A–D are representative of three repeats. (E) Model for how Sec16 and Sed4 function together in ERES localization. Sed4 interacts with itself to form at least a homodimer and is preferentially distributed to the high-curvature areas of the ER such as the tubules and edges of the sheets. Sec16 interacts with Sed4 to localize to ERES, which leads to concentration of Sed4 at ERES. Sec12 does not interact with Sec16 and is distributed to the entire ER, but is excluded from ERES.

*P. pastoris* Sec12 (PpSec12) has an extended luminal domain similar to Sed4 and has been reported to interact with itself to form a homodimer through the luminal domain (Soderholm et al., 2004). To examine the interaction of Sed4 with itself, we co-expressed Sed4–V5 with Sed4–Flag in cells and performed co-immunoprecipitation assays using anti-Flag antibody (Fig. 8A). Co-precipitation of Sed4–V5 was detected with Sed4–Flag but not with a control vector. These results clearly indicate that Sed4 interacts with itself. We then asked which domain of Sed4 is engaged in self-interaction by employing Flag-tagged versions of the chimera constructs described in Fig. 3A. Co-immunoprecipitation assays revealed that Sed4^12TM^–Flag interacts with Sed4–V5 and Sed4–Flag, but compared with these constructs, Sed4^12C^–Flag and Sed4^12L^–Flag showed considerably weaker interactions with Sed4–V5. These results suggest that the self-interaction of Sed4 requires both the cytosolic and luminal domains. This idea was confirmed by co-immunoprecipitation assays showing that the cytosolic domain-truncated Sed4^ΔC^–Flag and the luminal domain-truncated Sed4^ΔL^–Flag failed to interact with Sed4–V5 (Fig. S5A).

Next, we tested whether the self-interaction of Sed4 involves its interaction with Sec16. Sed4–V5 and Sed4–Flag were co-expressed in *sec16*Δ *sed4*Δ cells expressing Sec16 or Sec16^ΔS4B^. The co-precipitated Sed4–V5 with Sed4–Flag was observed at the same level in these cells with Sec16 or Sec16^ΔS4B^ (Fig. 8B), indicating that Sed4 self-interaction is independent of the interaction with Sec16.

A total of 55 O-mannosylation sites have been identified in Sed4 and were found to be evenly located in the luminal domain (Neubert et al., 2016). To examine the requirement of O-mannosylation for Sed4 function, we replaced all mannosylated residues with alanine residues to create an O-mannosylation-defective mutant, Sed4^ΔOM^. The co-immunoprecipitation assay showed that Sed4^ΔOM^–Flag did not interact with Sed4–V5 to the level of Sed4–Flag (Fig. 8C), suggesting that the self-interaction of Sed4^ΔOM^ is not as stable as that of Sed4. The self-interaction of PpSec12 has been previously reported to be coupled with its localization to the ERES (Soderholm et al., 2004). However, when expressed in *sed4*Δ cells, Sed4^ΔOM^–mUkG1 and Sec16–tdTomato were observed to be well concentrated at the ERES (Fig. 8D). These results indicate that O-mannosylation affects Sed4 self-interaction, but is not essential for the ERES localization of Sed4 and Sec16. This may suggest the possibility that unlike PpSec12, Sed4 does not associate self-interaction with ERES localization. However, we could not verify whether the Sed4^ΔOM^ self-interaction might be formed in the ER membrane but fragile enough to be disrupted in detergent-solubilized extracts. If so, such an unstable self-interaction of Sed4^ΔOM^ could potentially contribute to ERES localization.

To further test the possibility, we employed luminal domain-truncated Sed4^Δ544L^ and Sed4^Δ942L^ mutants. Based on the results shown in Fig. S4, these mutants were expected to exhibit different distribution patterns. Sed4–V5 was found to be normally co-precipitated with both Sed4^Δ544L^–Flag and Sed4^Δ942L^–Flag (Fig. S5B). In addition, fluorescence microscopy revealed that Sec16-tdTomato was properly distributed to ERES in *sed4*Δ cells expressing Sed4^Δ544L^–mUkG1 and Sed4^Δ942L^–mUkG1. However, Sed4^Δ544L^–mUkG1 was found to be distributed throughout the ER but not to the ERES, whereas Sed4^Δ942L^–mUkG1 exhibited concentration at the ERES to the same level as Sed4–mUkG1 (Fig. S5C). These findings suggest that self-interaction and ERES localization are independently mediated by the luminal domain of Sed4.

## DISCUSSION

In *S. cerevisiae*, Sec16 has been characterized as an initial component assembled at the ERES or specific ER subdomains that eventually become the ERES. The mechanism by which Sec16 localizes to the ERES remains unclear. *S. cerevisiae* harbors Sec12 and the Sec12-like protein Sed4 (Hardwick et al., 1992). Sec12 has been reported to be essential for COPII vesicle formation but is dispensable for ERES localization of Sec16 (Okamoto et al., 2012). Sed4 has been identified as the binding partner of Sec16 a quarter century ago, but the significance and mechanism underlying its interaction with Sec16 were yet to be elucidated (Gimeno et al., 1995). This present study sheds light on this long-standing problem and leads us to the conclusion that Sed4 mediates Sec16 localization to ERES. However, we showed that the absence of Sed4 does not completely cause dispersion of Sec16 but still allows Sec16 to be partially present at the ERES. These results indicate that Sed4 is necessary, but not sufficient, for Sec16 localization, suggesting that an additional determinant exists for the ERES localization of Sec16. In some species, Sec16 has been reported to carry domains required for ERES localization (Sprangers and Rabouille, 2015). The ERES localization of mammalian Sec16 is suggested to require the ERES localization domain and its interaction with TANGO1 (Maeda et al., 2017). Similarly, in *S. cerevisiae*, to complete assembly at the ERES, Sec16 might utilize the ERES localization domain in addition to the Sed4-binding site. Although the ERES localization domain has not yet been determined in *S. cerevisiae*, reconstitution experiments have demonstrated that purified *S. cerevisiae*-derived Sec16 is able to bind to lipid membranes without the presence of additional components (Supek et al., 2002; Yorimitsu and Sato, 2012).

The next question was whether Sed4 localized to the ERES. In a previous study, Sed4 was not observed at the ERES in chemically fixed cells expressing Sed4–HA from a *2 µ* plasmid by immunofluorescence microscopy (Gimeno et al., 1995). Our present study clearly demonstrates Sed4 localization to the ERES. Sed4 was simultaneously found in the general ER, indicating that Sed4 can be distributed in two pools. To understand the mechanisms underlying the differential distribution of Sed4 to the ERES, we took advantage of the differences between Sed4 and Sec12 regarding the localization, function and interaction with Sec16. Our analysis of Sed4–Sec12 chimeric constructs revealed the requirement of both cytosolic and luminal domains for ERES localization of Sed4. In a previous study, using a similar approach with *S. cerevisiae*-*P. pastoris* Sec12 chimera, ERES localization of PpSedc12 was shown to require its cytosolic and luminal domains (Soderholm et al., 2004). The cytosolic domain was hypothesized to bind a partner component that could target PpSec12 to the ERES. In a later study, Sec16 was identified as a binding and ERES-targeting partner (Montegna et al., 2012). However, it was unclear whether the interaction of PpSec12 with Sec16 is directly linked to the ERES localization of PpSec12. We discovered that Sec16 binds to β-propeller blades #1–3 of Sed4 but not to those of Sec12, which provides detailed insight into how Sec16 acts in ERES localization of Sed4. This is based on observations of ERES localization of Sed4^12C4b1-3^, and vice versa, with no ERES localization of Sed4^12C^ and Sed4^12C4b3-5^. Additionally, Sed4 was not concentrated at the ERES in *sec16*Δ cells expressing Sec16^ΔS4B^. These results provide direct evidence that Sed4 interacts with Sec16 through its cytosolic domain, which subsequently recruits Sed4 to the ERES. Consequently, Sec16 and Sed4 mutually localize and function at the ERES.

Sec12 has a catalytically critical K-loop in the region of blades #1–3 (McMahon et al., 2012). Because of its similar sequence to the Sec12 cytosolic domain, Sed4 is also predicted to possess a K-loop (Schlacht and Dacks, 2015). However, our results show that Sed4^12C4b1-3^ can mediate Sed4 functions but not those of Sec12, suggesting that the Sed4-derived K-loop carries a different function and does not function as a GEF. This is consistent with biochemical results showing that Sed4 has no GEF activity (Kodera et al., 2011; Saito-Nakano and Nakano, 2000). In contrast, Sed4^12C4b3-5^ retains the Sec12-derived K-loop, but cannot mediate Sed4 nor Sec12 functions. In the crystal structure (PDB: 6X90) of the Sec12–Sar1 complex, the region of blades #3–5 of Sec12 contains multiple residues that participate in the Sec12–Sar1 interface. Mutations in these residues were shown to inhibit GEF activity (Joiner and Fromme, 2021). We predict that because Sed4 and Sec12 bind to Sar1 in a different manner, Sed4^12C4b3-5^ has a disturbed interface that affects the ability for the protein to bind Sar1, causing GEF inactivation.

Regardless of the requirement of the luminal domain of Sed4 for ERES localization, there was no sequence similarity in this domain, excluding the HDEL sequence, between Sed4 and PpSec12. We found that this characteristic HDEL sequence was not required for the ERES localization of Sed4. The HDEL sequence is known to act as an ER retention signal. However, the mUkG1 fusion and HDEL-truncated constructs were localized to the ER and ERES. The same phenomenon was observed for PpSec12 (Soderholm et al., 2004). We cannot rule out the possibility that the HDEL sequence of these proteins plays a role in processes other than the COPII transport system.

Further investigations of the luminal domain-related mutants determined that Sed4 can show self-interaction, in addition to its ERES localization. We found that the three mutants Sed4^12L^, Sed4^Δ544L^ and Sed4^ΔOM^ behave differently in self-interaction and ERES localization, which suggests that these two processes could be controlled by different signals in the luminal domain. When these mutants were classified accordingly, Sed4^Δ544L^ retained the signal for self-interaction but lacked the signal for ERES localization, Sed4^ΔOM^ carried the signal for ERES localization but lost the signal for stable self-interaction, and Sed4^12L^ lost both signals for these processes. Because they enable Sec16 to localize normally to the ERES, these Sed4 mutants probably interact with Sec16. This suggests that the self-interaction or ERES localization of Sed4 is not essential for the ERES localization of Sec16.

The self-interaction of Sed4 could be regulated by the region of amino acid residues 370–553 of the luminal domain of Sed4, which corresponds to the region immediately downstream of the transmembrane domain. This is supported by the observation that Sed4 interacts with Sed4^Δ544L^ but not with Sed4^ΔC^ or Sed4^12L^. Similarly, the luminal domain of PpSec12 has been suggested to carry a signal to mediate its self-interaction in the region proximal to the transmembrane domain (Soderholm et al., 2004). Although its role is unknown, this region of PpSec12 is relatively rich in basic residues. It should be noted that there is no similarity in these regions between Sed4 and PpSec12. Instead, we discovered that O-mannosylation is involved in Sed4 self-interaction, suggesting that Sed4 undergoes O-mannosylation to regulate its self-interaction status. In addition to Sed4, proteins involved in the early secretory pathway, such as Sec12 and cargo receptors, are known to possess an O-mannosylated luminal domain in *S. cerevisiae* (Neubert et al., 2016). However, the physiological function of O-mannosylation is not well understood. This study provides the first evidence for a link between protein function and O-mannosylation in the COPII transport system.

We found that upon loss of interaction with Sec16, Sed4 is redistributed from the ERES exclusively to the tubules and edges of the sheets of the ER, but not to the sheets. Truncation of the luminal domain was shown to release Sed4 from this exclusive distribution and uniformly disperse it to the ER. These findings suggest that, to be concentrated at the ERES by Sec16, Sed4 needs to be pooled in the high-curvature areas of the ER. Based on this idea, the signal required for ERES localization of Sed4 in the luminal domain is predicted to correspond to the signal that triggers its exclusive distribution to high-curvature areas. Given that *P. pastoris* and mammalian Sec12 interact with Sec16 to localize to the ERES (Montegna et al., 2012), it would be interesting to know whether loss of the interaction with Sec16 causes redistribution of Sec12 from the ERES to high-curvature areas of the ER in other species.

There are two possible mechanisms through which the luminal domain of Sed4 mediates the distribution. One possibility is that the luminal domain of Sed4 has the ability to directly sense the curvature of the membranes. If so, it could recognize the convex curvature in the lumen. The inverse- Bin-Amphiphysin-Rvs (BAR) domain is known to sense and/or induce convex membrane curvature. All known I-BAR proteins are soluble, including the putative I-BAR protein Ivy1 in *S. cerevisiae*, and do not share sequence similarity (Itoh et al., 2016). Whether Sed4 senses a convex membrane in a manner similar to that of the I-BAR domain is unclear. The second possibility is that the luminal domain has binding partners that recruit Sed4 to high-curvature areas. ER-shaping proteins, such as reticulons and Yop1, are well known to localize to high-curvature areas (Voeltz et al., 2006). However, these proteins should be excluded from the candidates of the binding partners because Sed4 localization to high-curvature areas was observed in *rtn1*Δ *rtn2*Δ *yop1*Δ cells.

Altogether, we propose a model for how Sec16 and Sed4 function interdependently at the ERES (Fig. 8E). Sec16 interacts with Sed4 and subsequently localizes to ERES. In addition to its interaction with Sed4, Sec16 also requires an endogenous ERES localization domain to complete ERES localization. Sec16 can then concentrate Sed4 at the ERES. For the concentration, Sed4 needs to be preliminarily accumulated in the high-curvature areas of the ER, which is mediated by the action of its luminal domain. Sec12 does not interact with Sec16 and is present throughout the entire ER, but is excluded from the ERES as reported previously (Okamoto et al., 2012).

Finally, the question arises as to whether Sec16 and Sed4 function together as GTPase regulatory partners at the ERES. In the model for GTPase regulation, Sec16 organizes COPII assembly by inhibiting Sec31-dependent Sec23 GAP activity against Sar1 (Kung et al., 2012; Yorimitsu and Sato, 2012), and Sed4 facilitates the formation of cargo-loaded vesicles by activating the Sar1 GTPase and Sec23 GAP (Kodera et al., 2011). Two distinct binding sites for Sed4 and Sec23 were found to be located in the 360-amino-acid region of the C-terminal end of Sec16, and the regions of Sed4 that mediate Sec16 binding contain the K-loop, although Sed4 is not determined to engage the K-loop in GTPase regulation. It is possible that Sec16 controls the GTPase regulatory activity of coat subunits and Sed4 in the same complex at the ERES to promote the efficient formation of cargo-loaded vesicles. Future biochemical studies are needed to address this possibility.

## MATERIALS AND METHODS

### Strains and plasmids

The yeast strains used in this study are listed in Table S1. All strains are isogenic to YPH500 (Sikorski and Hieter, 1989), except for PJ69-4A and MBY10-7A. The strains were grown at 30°C in YPD medium (2% peptone, 1% yeast extract and 2% glucose) or synthetic medium (0.67% yeast nitrogen base without amino acids and 2% glucose, supplemented with appropriate nutrients) (0.5% casamino acids, 0.002% adenine, 0.002% uracil, 0.002% histidine, 0.003% leucine, 0.002% tryptophan, 0.003% lysine, 0.002% methionine). Gene deletion was performed using standard homologous recombination methods (Longtine et al., 1998). Counter selection against *URA3*-based plasmids was performed on synthetic medium plates containing 0.1% 5-fluoroorotic acid (Fujifilm Wako Chemicals).

The plasmids used in this study are listed in Table S2. Plasmids were constructed using the DNA Ligation Kit Mighty Mix (TAKARA Bio) or NEBuilder HiFi DNA Assembly Master Mix (NEB). PCR-based gene amplification was performed using Phusion DNA polymerase (NEB). Artificially synthesized DNA fragment coding yeast codon-optimized mScarlet, as shown previously, was purchased from Europhins Genomics. Artificially synthesized DNA fragments coding for the luminal domain of Sed4^ΔOM^ were purchased from IDT and assembled using NEBuilder HiFi DNA Assembly Master Mix. Except for those on the yeast two-hybrid plasmids, all genes were expressed under the control of their own promoters on the *CEN* plasmid. For the yeast two-hybrid assay, genes were cloned into pGBDU-C1 to be expressed as Gal4 DNA-binding domain-fused proteins, or into pGAD-C1 to be expressed as Gal4 activation domain-fused proteins (James et al., 1996).

### Yeast two-hybrid assay

Yeast two-hybrid strains were transformed with pGBDU-C1- and pGAD-C1-based constructs and transformants were selected on synthetic medium plates lacking leucine and uracil. To test protein interactions, transformants were incubated on synthetic medium plates lacking leucine and uracil, or lacking histidine, leucine and uracil at 30°C for 4 days.

### Co-immunoprecipitation assay

For co-immunoprecipitation between Sed4–V5 and Sec16–HA, cells were grown to mid-log phase and 500 OD_600_ units of cells were collected. After washing with water, the cells were resuspended in lysis buffer (25 mM HEPES pH 8.0, 50 mM NaCl, 1 mM EDTA, 5% glycerol) with 2× EDTA-free protease inhibitor cocktail (Roche) and disrupted by vigorous vortexing with glass beads at 4°C. The cell lysates were mixed with an equal volume of lysis buffer containing 4% octylglucoside (Dojindo) and incubated on ice for 20 min. After insolubilized materials were removed by centrifugation at 10,000 ***g*** for 5 min at 4°C, the supernatant was transferred to a new tube and mixed with 1* *µl of mouse anti-V5 (MCA1360; Bio-Rad) or 0.5* *µl of mouse anti-HA antibody (901514; Biolegend). After 1 h of incubation at 4°C, 10* *µl of Protein A–Sepharose beads (GE Healthcare) equilibrated with lysis buffer containing 1% octylglucoside were added and incubated for 1 h at 4°C. The collected beads were washed three times with lysis buffer containing 1% octylglucoside and the proteins were eluted by boiling in SDS sample buffer. The eluted proteins were separated by SDS-PAGE on 6% polyacrylamide gels prepared with WIDE RANGE Gel Preparation Buffer (Nacalai Tesque), followed by immunoblotting with mouse anti-HA (1:10,000, 901514; Biolegend) and anti-V5 antibodies (1:25,000, MCA1360; Bio-Rad).

For co-immunoprecipitation between Sed4–V5 and Sed4–Flag, cells were grown to mid-log phase, and 200 OD_600_ units of cells were collected. After washing with water, the cells were resuspended in lysis buffer (25 mM HEPES pH 8.0, 150 mM NaCl, 1 mM EDTA, 5% glycerol) with 2× EDTA-free protease inhibitor cocktail and disrupted by vigorous vortexing with glass beads at 4°C. The cell lysates were mixed with an equal volume of lysis buffer containing 4% octylglucoside and incubated on ice for 20 min. After the insolubilized materials were removed by centrifugation at 10,000 ***g*** for 5 min at 4°C, the supernatant was transferred to a new tube and mixed with 10* *µl of Protein A–Sepharose beads equilibrated with lysis buffer containing 2% octylglucoside. After 1 h of incubation at 4°C, beads were removed by centrifugation (10,000 ***g*** for 1 min at 4°C), and the supernatant was transferred to a new tube and mixed with 1* *µl of mouse anti-DDDDK antibody (M185-3L; MBL) and 10* *µl of fresh Protein A–Sepharose beads equilibrated with lysis buffer containing 1% octylglucoside. After 1 h of incubation at 4°C, the collected beads were washed six times with lysis buffer containing 1% octylglucoside. The beads were transferred to a new tube and washed once with a lysis buffer containing 1% octylglucoside. The proteins were eluted by boiling in SDS sample buffer and separated by SDS-PAGE on 6% polyacrylamide gels prepared with WIDE RANGE Gel Preparation Buffer, followed by immunoblotting with mouse anti-DDDDK (1:25,000, M185-3L; MBL) and anti-V5 antibodies. All images of uncropped blots were shown in Fig. S6.

### Fluorescence microscopy

Cells were incubated at 30°C in synthetic medium and grown to mid log phase. Cells were visualized using an Olympus IX71 microscope (Olympus) equipped with a CSU10 spinning-disk confocal scanner (Yokogawa Electric Corporation), as described previously (Yorimitsu and Sato, 2012). Images were captured using an electron-multiplying charge-coupled device camera (iXon, DV897; Andor Technology). Photoshop (Adobe) and ImageJ (National Institutes of Health) were used to prepare the images for figure preparation.

### Quantification and statistical analysis

ImageJ was used to quantify the fluorescence intensity of cytosolic versus ERES-localized Sec16–tdTomato, Sec31–mCherry and Sec23–mUkG1 proteins. The intensity values of the background, cell cytosol, and ERES were obtained from the fluorescence microscopy images of wild-type and *sed4*Δ cells expressing each protein. The background value was subtracted from the values obtained from the cytosolic and ERES signals, and the ERES value was divided by that of the cytosol. At least 40 cells were monitored in images obtained from three independent experiments.

Line-scan analysis was performed using ImageJ software. The intensity values of Sec71–EGFP, Sec12–mScarlet, Sed4–mScarlet and Sed4^12C^–mScarlet, including its derivative mutants, were quantified along the potted lines. The background value was subtracted from that obtained for each protein, and using these subtracted values, normalized to the maximum value; intensity profile plots were generated using Microsoft Excel (Microsoft Corporation).

All statistical analyses were performed using Microsoft Excel. All data are presented as the mean±s.d. *P*-values were calculated using an unpaired two-tailed *t*-test.

## Supplementary Material

10.1242/joces.261094_sup1 Supplementary information
