# Supplementary material for: Sec16 and Sed4 interdependently function as interaction and localization partners at ER exit sites
Source: J Cell Sci. 2023 May 9;136(9):jcs261094. doi: 10.1242/jcs.261094 (PMC10184828; doi:10.1242/jcs.261094)
Supplement: Supplementary information [file joces-136-261094-s1.pdf]

Fig. S1

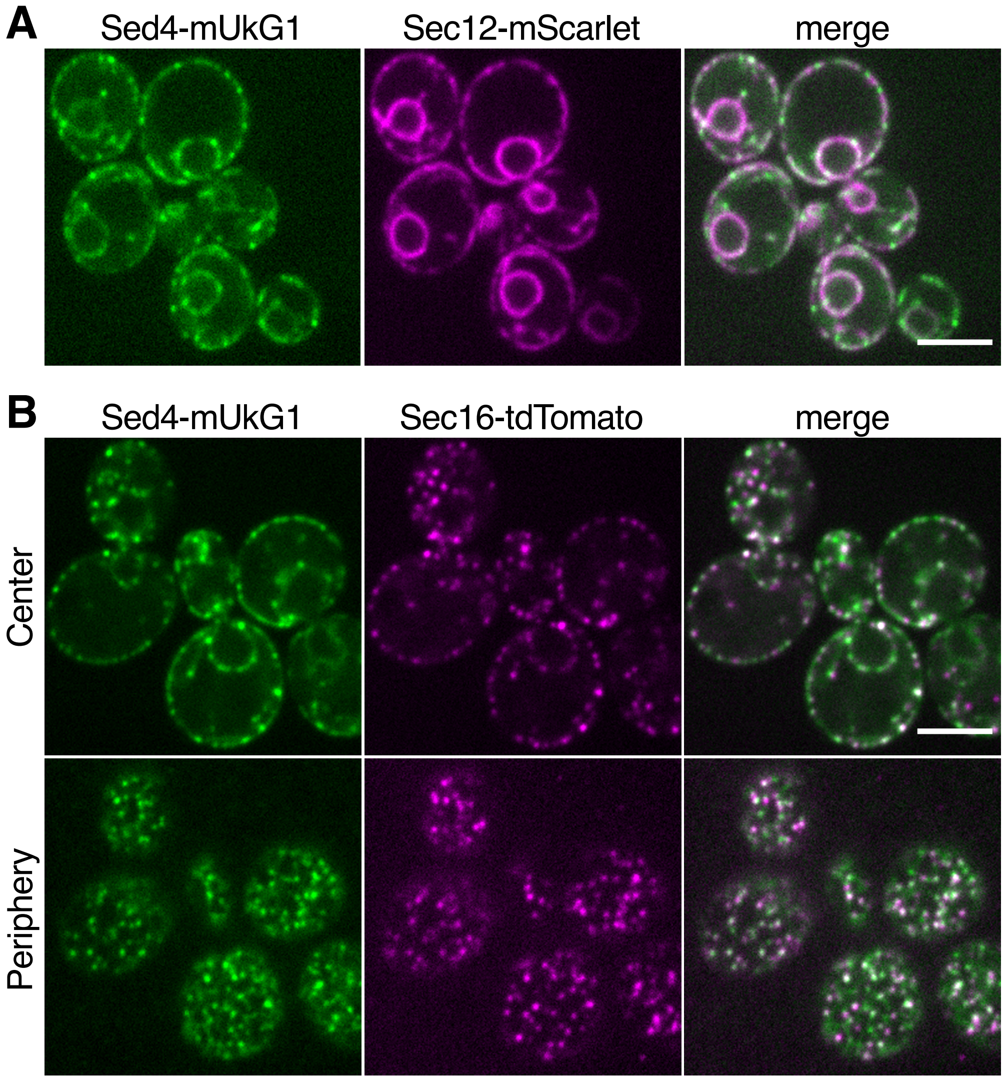

Fig. S1. Sed4 is concentrated at ERES. (A) Wild-type cells expressing Sed4-mUkG1 with Sec12-mScarlet were grown to a mid-log phase and observed by fluorescence microscopy. Scale bar: 4 µm. **(B)** *sed4*Δ *sec16*Δ cells expressing Sec16-tdTomato with Sed4-mUkG1 were grown to a mid-log phase and observed by fluorescence microscopy. The ER and ERES were visualized by focusing on either the center or the periphery of the cell. Scale bar: 4 µm. Images are representative of 3 repeats.

Fig. S2

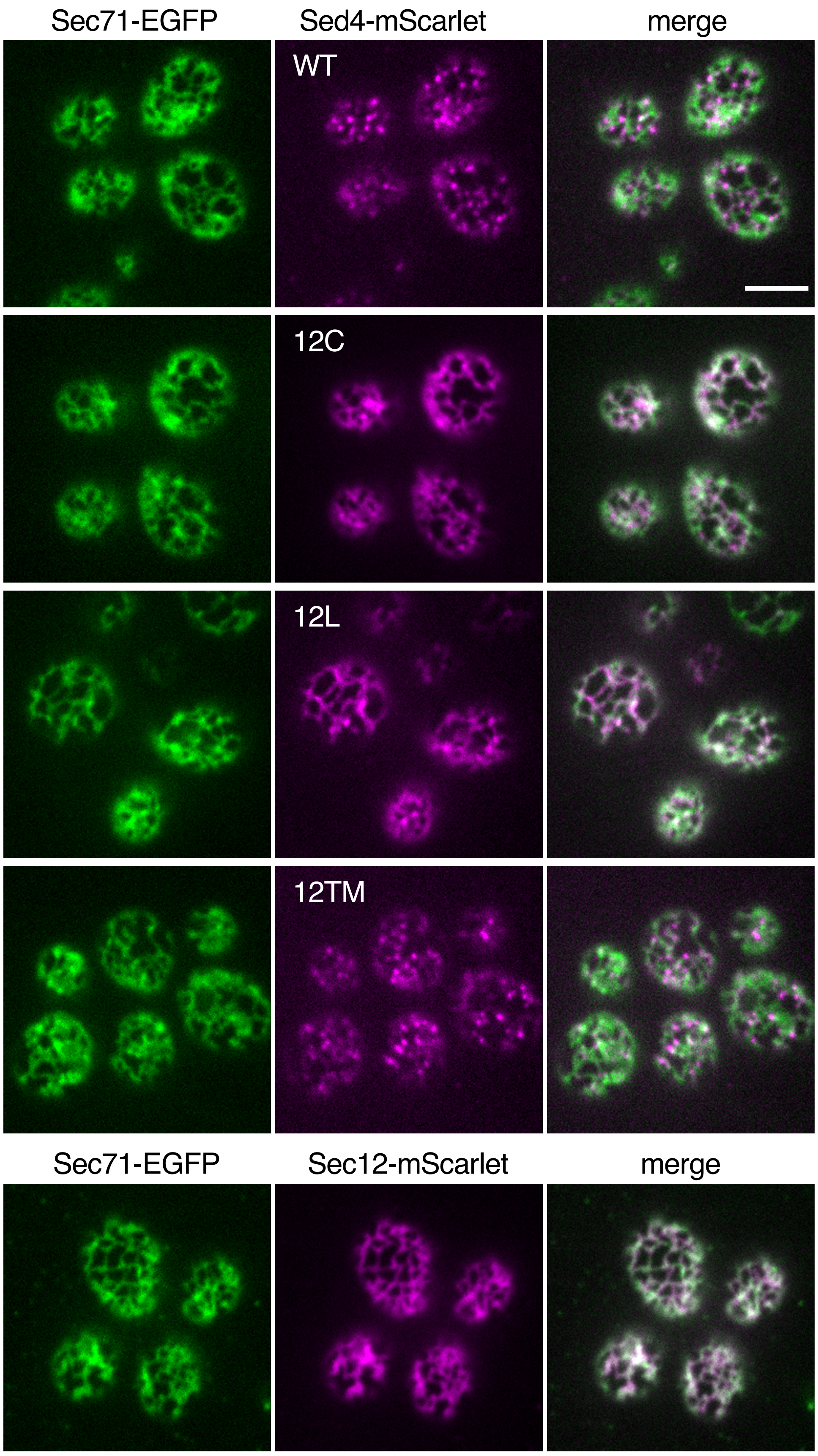

Fig. S2. Sed4^{12C} and Sed4^{12L} are distributed throughout the ER without accumulation at ERES and Sed4^{12TM} is concentrated at ERES. Wild-type cells expressing Sec71-EGFP with Sed4-mScarlet, Sed4^{12C}-mScarlet, Sed4^{12L}-mScarlet, Sed4^{12TM}-mScarlet or Sec12-mScarlet were grown to a mid-log phase and observed by fluorescence microscopy. The ER and ERES were visualized by focusing on the periphery of the cell. Scale bar: 4 μm. Images are representative of 3 repeats.

Journal of Cell Science • Supplementary information

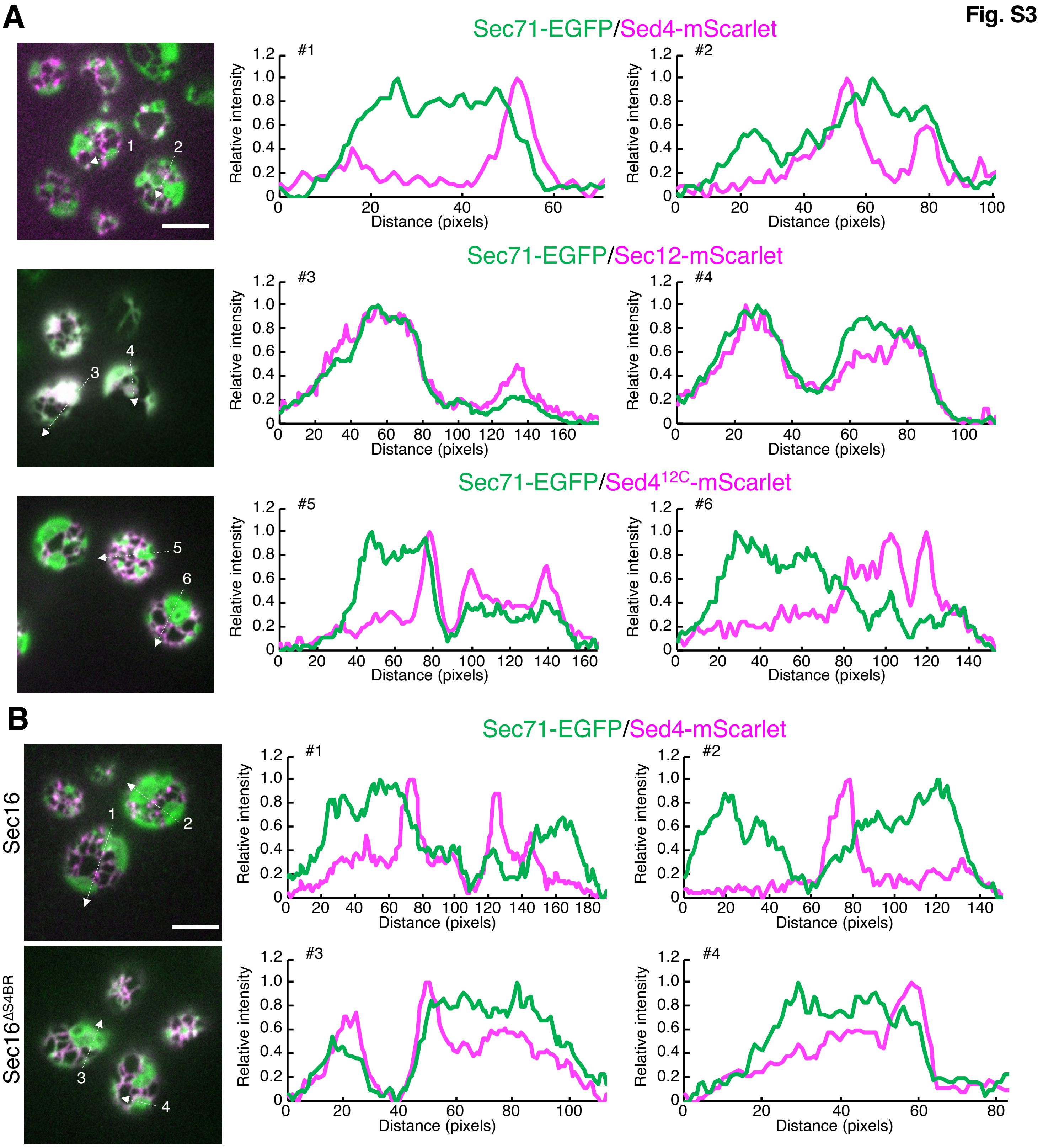

Fig. S3. Preferential localization of Sed4 to the tubules and edges of the sheets of the ER without interaction with Sec16 (A) *rtn1*Δ *rtn2*Δ *yop1*Δ cells expressing Sec71-EGFP with Sed4-mScarlet, Sed4^{12C}-mScarlet, or Sec12-mScarlet were grown to a mid-log phase and observed by fluorescence microscopy. The ER and ERES were visualized by focusing on the periphery of the cell. Scale bar: 4 µm. **(B)** *rtn1*Δ *rtn2*Δ *yop1*Δ *sec16*Δ cells expressing Sec71-EGFP and Sed4-mScarlet with Sec16 or Sec16$^{\Delta S4B}$ were grown to a mid-log phase and observed by fluorescence microscopy. The ER and ERES were visualized by focusing on the periphery of the cell. In the right panels of (A) and (B), white arrows indicate the sheet regions of the ER. Line-scan analysis was carried out in the area indicated by a white dashed arrow in the merged images, and profile plots of the normalized intensity are shown. Scale bar: 4 µm. Images are representative of 3 repeats.

Fig. S4

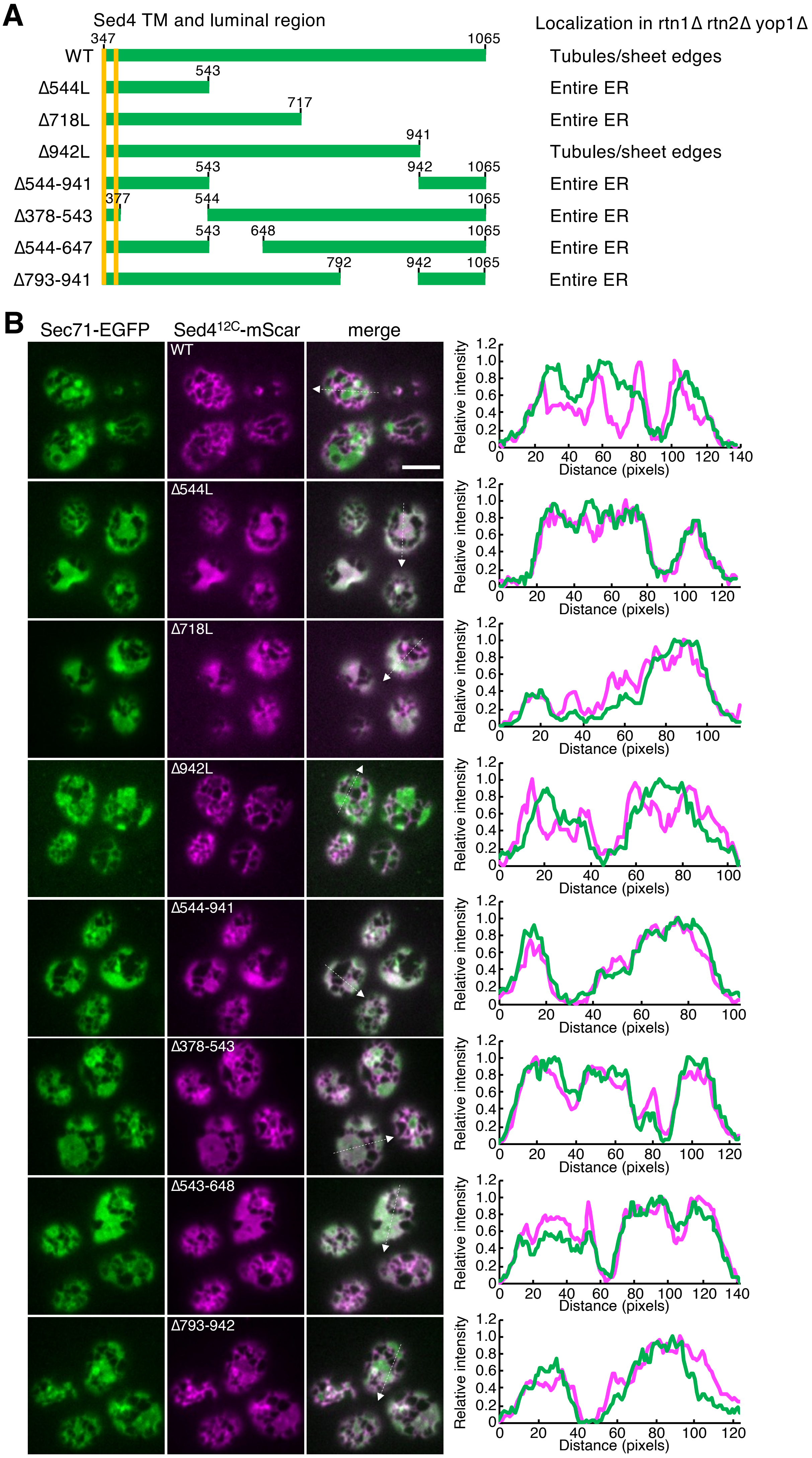

Journal of Cell Science • Supplementary information

Fig. S4. Entire or multiple regions of the luminal region are required for preferential localization of Sed4 to the high-curvature areas of the ER (A) Schematic diagrams of the truncated region of the luminal domain of $Sed4^{12C}$. The area of the ER where each truncation mutant localizes is shown on the left, which is based on the images obtained in (B). **(B)** *rtn1*Δ *rtn2*Δ *yop1*Δ cells expressing Sec71-EGFP with $Sed4^{12C}$-mScarlet or its truncation construct indicated were grown to a mid-log phase and observed by fluorescence microscopy. In the right panels, line-scan analysis was carried out at the area indicated by a white dashed arrow in the merged images, and profile plots of the normalized intensity are shown. Scale bar: 4 μm. Images in B are representative of 3 repeats.

Fig. S5

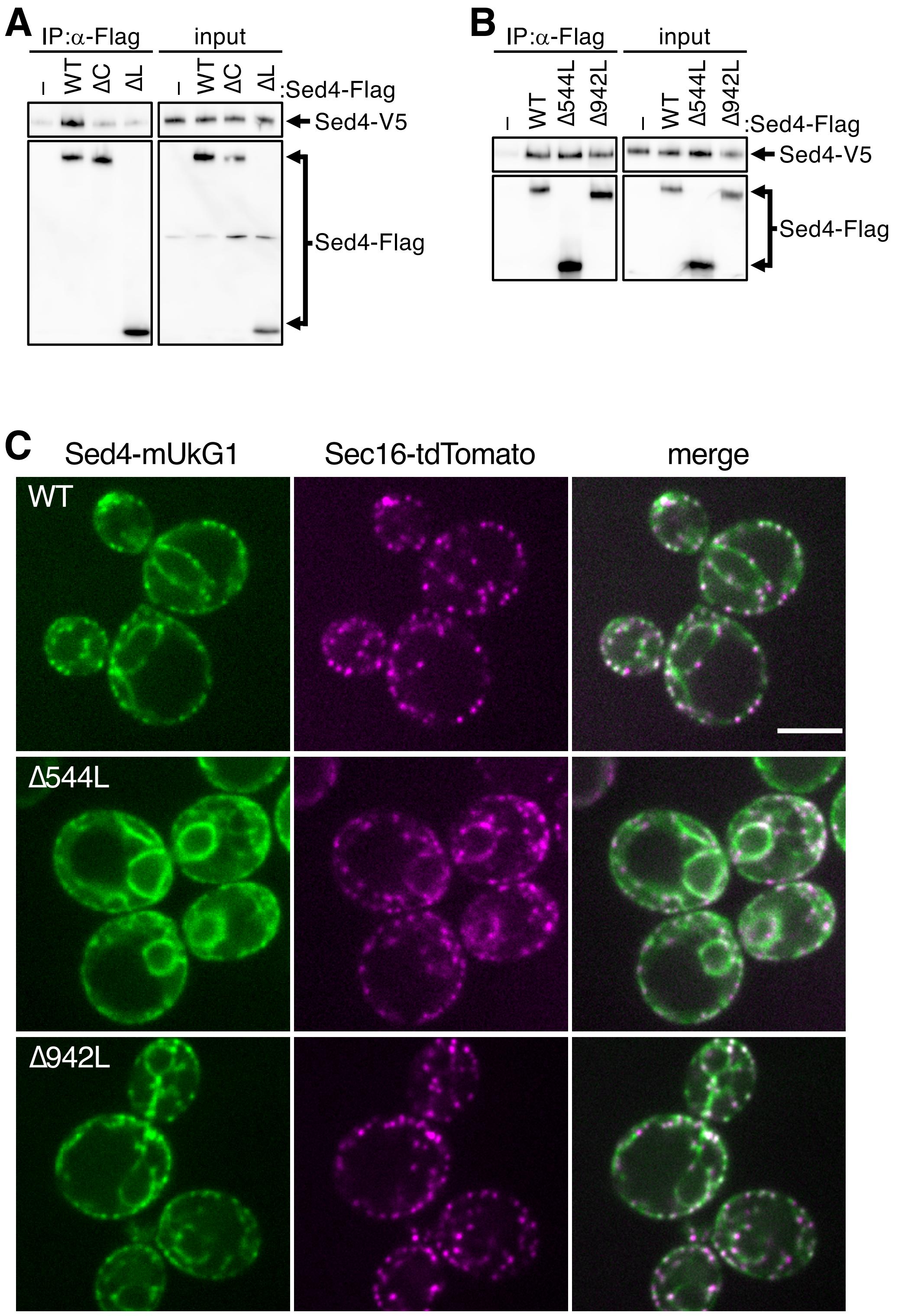

Fig. S5. Sed4 self-interaction that requires the cytosolic and luminal domains is not sufficient for ERES localization. (A) *sed4*Δ cells expressing Sed4-V5 with or without Sed4-Flag (WT), Sed4$^{\Delta C}$-Flag (ΔC), or Sed4$^{\Delta L}$-Flag (ΔL) were grown and collected at mid-log phase. After immunoprecipitation, precipitated proteins were analyzed, as described in Fig. 8A. Input, 0.05%. **(B)** *sed4*Δ cells expressing Sed4-V5 with or without Sed4-Flag (WT), Sed4$^{\Delta 544L}$-Flag (Δ544L), or Sed4$^{\Delta 942L}$-Flag (Δ942L) were grown and collected at mid-log phase. After immunoprecipitation, precipitated proteins were analyzed as described in Fig. 8A. Input, 0.05%. **(C)** *sed4*Δ *sec16*Δ cells expressing Sec16-tdTomato with Sed4-mUkG1 (WT), Sed4$^{\Delta 544L}$-mUkG1 (Δ544L), or Sed4$^{\Delta 942L}$-mUkG1 (Δ942L) were grown to a mid-log phase and observed by fluorescence microscopy. Scale bar: 4 µm. Images are representative of 3 repeats.

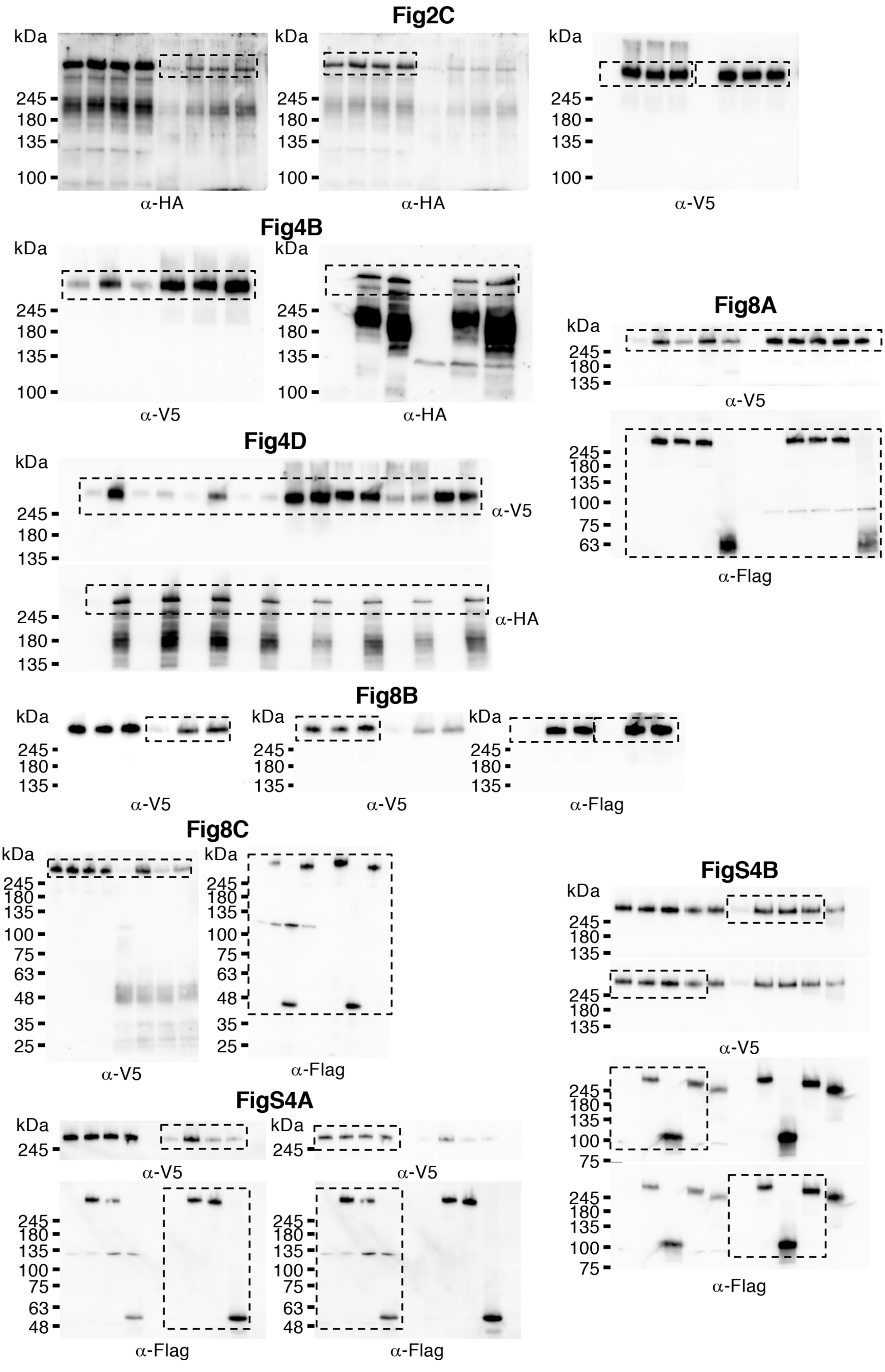

Fig. S6. Uncropped blot images.

Journal of Cell Science • Supplementary information

Table S1. Yeast strains used in this study

Name	Genotype	Figure	Source
MBY10-7A	MATa *leu2-3,112 ura3-52 his3 his4 trp1-289 suc2-Δ9 gal2 sec12-4*	6B	Kakoi *et al.*, 1996
PJ69-4A	MATa *trp1-901 leu2-3,112 ura3-52 his3-200 gal4Δ gal80Δ LYS2::GAL1-HIS3 GAL2-ADE2 met2::GAL7-lacZ*	4AC	James *et al.*, 1996
YTY044	MATα *ura3-52 lys2-801 ade2-101 trp1-Δ63 his3-Δ200 leu2-Δ1 ADE2*	1AB, S1A	This study
YTY049	MATα *ura3-52 lys2-801 ade2-101 trp1-Δ63 his3-Δ200 leu2-Δ1 ADE2 sec16Δ::KAN* pTYY48	1AB, 5A	Yorimitsu and Sato, 2020
YTY049-ΔS4B	MATα *ura3-52 lys2-801 ade2-101 trp1-Δ63 his3-Δ200 leu2-Δ1 ADE2 sec16Δ::KAN* pTYY48$^{\Delta S4B}$	5A	This study
YTY049-L1089P	MATα *ura3-52 lys2-801 ade2-101 trp1-Δ63 his3-Δ200 leu2-Δ1 ADE2 sec16Δ::KAN* pTYY48^{L1089P}	2D, 5A, 6A	This study
YTY195	MATα *ura3-52 lys2-801 ade2-101 trp1-Δ63 his3-Δ200 leu2-Δ1 ADE2 sed4Δ::HIS3*	1AB, 7A, 8AC, S4AB	This study
YTY207	MATα *ura3-52 lys2-801 ade2-101 trp1-Δ63 his3-Δ200 leu2-Δ1 ADE2 rtn1Δ::NAT rtn2Δ::HIS3 yop1Δ::KAN*	7A, S2A, S3B	This study
YTY370	MATα *ura3-52 lys2-801 ade2-101 trp1-Δ63 his3-Δ200 leu2-Δ1 ADE2 sec16Δ::KAN* pTYY55	1AB,	This study
YTY370-ΔS4B	MATα *ura3-52 lys2-801 ade2-101 trp1-Δ63 his3-Δ200 leu2-Δ1 ADE2 sec16Δ::KAN* pTYY55$^{\Delta S4B}$		This study
YTY429	MATα *ura3-52 lys2-801 ade2-101 trp1-Δ63 his3-Δ200 leu2-Δ1 ADE2 sec16Δ::KAN sed4Δ::HIS3* pTYY55	2AB, 3BC, 5BC, 6CD, 8D, S1B, S4C	This study
YTY429-ΔS4B	MATα *ura3-52 lys2-801 ade2-101 trp1-Δ63 his3-Δ200 leu2-Δ1 ADE2 sec16Δ::KAN sed4Δ::HIS3* pTYY55$^{\Delta S4B}$	5BC	This study
YTY430	MATα *ura3-52 lys2-801 ade2-101 trp1-Δ63 his3-Δ200 leu2-Δ1 ADE2 sec16Δ::KAN sed4Δ::HIS3* pTYY48	2C, 4BD	This study
YTY430-ΔS4B	MATα *ura3-52 lys2-801 ade2-101 trp1-Δ63 his3-Δ200 leu2-Δ1 ADE2 sec16Δ::KAN sed4Δ::HIS3* pTYY48$^{\Delta S4B}$	4B, 5A	This study
YTY430-L1089P	MATα *ura3-52 lys2-801 ade2-101 trp1-Δ63 his3-Δ200 leu2-Δ1 ADE2 sec16Δ::KAN sed4Δ::HIS3* pTYY48^{L1089P}	2D, 5A, 6A	This study
YTY437	MATα *ura3-52 lys2-801 ade2-101 trp1-Δ63 his3-Δ200 leu2-Δ1 ADE2 sec16Δ::KAN sed4Δ::HIS3* pSec16-tdTomato (315)	8B	This study
YTY437-ΔS4B	MATα *ura3-52 lys2-801 ade2-101 trp1-Δ63 his3-Δ200 leu2-Δ1 ADE2 sec16Δ::KAN sed4Δ::HIS3* pSec16$^{\Delta S4B}$-tdTomato (315)	8B	This study
YTY439	MATα *ura3-52 lys2-801 ade2-101 trp1-Δ63 his3-Δ200 leu2-Δ1 ADE2 sec16Δ::KAN rtn1Δ::HIS3 rtn2Δ::HPH yop1Δ::NAT* pTYY55	5D	This study
YTY439-ΔS4B	MATα *ura3-52 lys2-801 ade2-101 trp1-Δ63 his3-Δ200 leu2-Δ1 ADE2 sec16Δ::KAN rtn1Δ::HIS3 rtn2Δ::HPH yop1Δ::NAT* pTYY55$^{\Delta S4B}$	5D	This study
YTY440	MATα *ura3-52 lys2-801 ade2-101 trp1-Δ63 his3-Δ200 leu2-Δ1 ADE2 sec16Δ::KAN rtn1Δ::HIS3 rtn2Δ::HPH yop1Δ::NAT* pSec16-HA (315)	5D7B, S2B	This study
YTY440-ΔS4B	MATα *ura3-52 lys2-801 ade2-101 trp1-Δ63 his3-Δ200 leu2-Δ1 ADE2 sec16Δ::KAN rtn1Δ::HIS3 rtn2Δ::HPH yop1Δ::NAT* pSec16$^{\Delta S4B}$-HA (315)	7B, S2B	This study

Table S2. Plasmids used in this study

Name	Vector	Description	Figure	Source
pRS314		*TRP1*, *CEN*	8ABC, S4AB	Sikorski and Hieter, 1989
pRS316		*URA3*, *CEN*	2CD, 6AB	Sikorski and Hieter, 1989
pQR316-Sec71-GFP	pRS316	$P_{SEC71}\text{-}SEC71\text{-}EGFP$	5D, 7AB, S2AB, S3B	Sato *et al.*, 2003
pSec12-mUkG1 (316)	pRS316	$P_{SEC12}\text{-}SEC12\text{-}mUkG1$	2AB, 3BC,	This study
pSec12-mScarlet (314)	pRS314	$P_{SEC12}\text{-}SEC12\text{-}mScarlet\text{-}I$	7A, S2A	This study
pSec12-mScarlet (316)	pRS316	$P_{SEC12}\text{-}SEC12\text{-}mScarlet\text{-}I$	S1A	This study
pSec23-mUkG1 (314)	pRS314	$P_{SEC23}\text{-}SEC23\text{-}mUkG1$	1AB	This study
pSec31-mCherry (314)	pRS314	$P_{SEC31}\text{-}SEC31\text{-}mCherry$	1AB	This study
pTYY48	pRS314	$P_{SEC16}\text{-}SEC16\text{-}3xHA$	2C, 4BC, 5A	Yorimitsu and Sato, 2012
pTYY48^{L1089P}	pRS314	$P_{SEC16}\text{-}SEC16^{L1089P}\text{-}3xHA$	2D, 5A, 6A	Yorimitsu and Sato, 2012
pTYY48$^{\Delta S4B}$	pRS314	$P_{SEC16}\text{-}SEC16^{\Delta 1856\text{-}1967}\text{-}3xHA$	4BD, 5A	This study
pTYY55	pRS314	$P_{SEC16}\text{-}SEC16\text{-}tdTomato$	1AB, 2AB, 3BC, 5BCD, 6CD, 8D, S1AB	This study
pTYY55$^{\Delta S4B}$	pRS314	$P_{SEC16}\text{-}SEC16^{\Delta 1856\text{-}1967}\text{-}tdTomato$	5BCD	This study
pSec16-HA (315)	pRS315	$P_{SEC16}\text{-}SEC16\text{-}3xHA$	7B, S2B	This study
pSec16$^{\Delta S4B}$-HA (315)	pRS315	$P_{SEC16}\text{-}SEC16^{\Delta 1856\text{-}1967}\text{-}3xHA$	7B, S2B	This study
pSec16-tdTomato (315)	pRS315	$P_{SEC16}\text{-}SEC16\text{-}tdTomato$	8B	This study
pSec16$^{\Delta S4B}$-tdTomato (315)	pRS315	$P_{SEC16}\text{-}SEC16^{\Delta 1856\text{-}1967}\text{-}tdTomato$	8B	This study
pSed4-mUkG1 (316)	pRS316	$P_{SED4}\text{-}SED4\text{-}mUkG1$	2AB, 3BC, 5BCD, 6CD, 8D, S1AB	This study
pSed4-mUkG1^{HDEL} (316)	pRS316	$P_{SED4}\text{-}SED4^{1\text{-}1061}\text{-}mUkG1^{HDEL}$	2AB, 3BC,	This study
pSed4$^{\Delta HDEL}$-mUkG1 (316)	pRS316	$P_{SED4}\text{-}SED4^{1\text{-}1061}\text{-}mUkG1$	2AB	This study
pSed4^{12C}-mUkG1 (316)	pRS316	$P_{SED4}\text{-}SEC12^{1\text{-}354}\text{-}SED4^{347\text{-}1065}\text{-}mUkG1$	3BC, 6CD	This study
pSed4^{12L}-mUkG1 (316)	pRS316	$P_{SED4}\text{-}SED4^{1\text{-}370}\text{-}SEC12^{379\text{-}471}\text{-}mUkG1$	3BC	This study
pSed4^{12TM}-mUkG1 (316)	pRS316	$P_{SED4}\text{-}SED4^{1\text{-}347}\text{-}SEC12^{357\text{-}372}\text{- }SED4^{365\text{-}1065}\text{-}mUkG1$	3BC	This study
pSed4$^{12C4b1\text{-}3}$-mUkG1 (316)	pRS316	$P_{SED4}\text{-}SEC12^{1\text{-}13}\text{-}SED4^{14\text{-}176}\text{-}SEC12^{182\text{-}354}\text{- }SED4^{347\text{-}1065}\text{-}mUkG1$	6CD	This study
pSed4$^{12C4b3\text{-}5}$-mUkG1 (316)	pRS316	$P_{SED4}\text{-}SEC12^{1\text{-}123}\text{-}SED4^{119\text{-}269}\text{-}SEC12^{275\text{-}354}\text{- }SED4^{347\text{-}1065}\text{-}mUkG1$	6CD	This study
pSed4$^{\Delta OM}$-mUkG1 (316)	pRS316	$P_{SED4}\text{-}SED4^{\Delta O\text{-}mannosylation}\text{-}mUkG1$	8D	This study
pSed4$^{\Delta 544L}$-mUkG1 (316)	pRS316	$P_{SED4}\text{-}SED4^{1\text{-}543}\text{-}mUkG1$	S4C	This study
pSed4$^{\Delta 942L}$-mUkG1 (316)	pRS316	$P_{SED4}\text{-}SED4^{1\text{-}941}\text{-}mUkG1$	S4C	This study
pSed4-mScarlet (314)	pRS314	$P_{SED4}\text{-}SED4\text{-}mScarlet\text{-}I$	7AB, S2AB	This study
pSed4^{12C}-mScarlet (314)	pRS314	$P_{SED4}\text{-}SEC12^{1\text{-}354}\text{-}SED4^{347\text{-}1065}\text{-}mScarlet\text{-}I$	7A, S2A, S3B	This study
pSed4$^{12C\Delta 544L}$-mScarlet (314)	pRS314	$P_{SED4}\text{-}SEC12^{1\text{-}354}\text{-}SED4^{347\text{-}543}\text{-}mScarlet\text{-}I$	S3B	This study
pSed4$^{12C\Delta 718L}$-mScarlet (314)	pRS314	$P_{SED4}\text{-}SEC12^{1\text{-}354}\text{-}SED4^{347\text{-}717}\text{-}mScarlet\text{-}I$	S3B	This study

Journal of Cell Science • Supplementary information

pSed4$^{12C\Delta942L}$-mScarlet (314)	pRS314	P_{SED4}-SEC12$^{1-354}$-SED4$^{347-941}$-mScarlet-I	S3B	This study
pSed4$^{12C\Delta544-941}$-mScarlet (314)	pRS314	P_{SED4}-SEC12$^{1-354}$-SED4$^{347-1065\Delta544-941}$-mScarlet-I	S3B	This study
pSed4$^{12C\Delta378-543}$-mScarlet (314)	pRS314	P_{SED4}-SEC12$^{1-354}$-SED4$^{347-1065\Delta378-543}$-mScarlet-I	S3B	This study
pSed4$^{12C\Delta544-647}$-mScarlet (314)	pRS314	P_{SED4}-SEC12$^{1-354}$-SED4$^{347-1065\Delta544-647}$-mScarlet-I	S3B	This study
pSed4$^{12C\Delta793-941}$-mScarlet (314)	pRS314	P_{SED4}-SEC12$^{1-354}$-SED4$^{347-1065\Delta793-941}$-mScarlet-I	S3B	This study
pSed4-V5 (316)	pRS316	P_{SED4}-SED4-3xV5	2CD, 4BD, 6AB	This study
pSed4-V5^{HDEL} (316)	pRS316	P_{SED4}-SED4$^{1-1061}$-3xV5^{HDEL}	2CD	This study
pSed4$^{\Delta HDEL}$-V5 (316)	pRS316	P_{SED4}-SED4$^{1-1061}$-3xV5	2CD	This study
pSed4^{12C}-V5 (316)	pRS316	P_{SED4}-SEC12$^{1-354}$-SED4$^{347-1065}$-3xV5	4BD, 6AB	This study
pSed4$^{12C4b1-3}$-V5(316)	pRS316	P_{SED4}-SEC12$^{1-13}$-SED4$^{14-176}$-SEC12$^{182-354}$-SED4$^{347-1065}$-3xV5	4BD, 6AB	This study
pSed4$^{12C4b3-5}$-V5 (316)	pRS316	P_{SED4}-SEC12$^{1-123}$-SED4$^{119-269}$-SEC12$^{275-354}$-SED4$^{347-1065}$-3xV5	4BD, 6AB	This study
pSed4-Flag (314)	pRS314	P_{SED4}-SED4-3xFlag	8ABC, S4AB	This study
pSed4^{12C}-Flag (314)	pRS314	P_{SED4}-SEC12$^{1-354}$-SED4$^{347-1065}$-3xFlag	8AB	This study
pSed4^{12L}-Flag (314)	pRS314	P_{SED4}-SED4$^{1-370}$-SEC12$^{379-471}$-3xFlag	8AB	This study
pSed4^{12TM}-Flag (314)	pRS314	P_{SED4}-SED4$^{1-347}$-SEC12$^{357-372}$- SED4$^{365-1065}$-3xFlag	8AB	This study
pSed4$^{\Delta N}$-Flag (314)	pRS314	P_{SED4}-SED4$^{340-1065}$-3xFlag	S4AB	This study
pSed4$^{\Delta C}$-Flag (314)	pRS314	P_{SED4}-SED4$^{1-369}$-3xFlag	S4AB	This study
pSed4$^{\Delta OM}$- Flag (314)	pRS314	P_{SED4}-SED4$^{\Delta O\text{-}mannosylation}$-3xFlag	8C	This study
pSed4$^{\Delta544L}$- Flag (314)	pRS314	P_{SED4}-SED4$^{1-543}$-3xFlag	S4B	This study
pSed4$^{\Delta942L}$- Flag (314)	pRS314	P_{SED4}-SED4$^{1-941}$-3xFlag	S4B	This study
pGAD-C1		P_{ADH1}-AD (GAL4 activation domain), LEU2, 2μ	4AC	James *et al*., 1996
pGBDU-C1		P_{ADH1}-BD (GAL4 DNA binding domain), URA3, 2μ		James *et al*., 1996
pGAD-Sec16$^{1639-2195}$	pGAD-C1	P_{ADH1}-AD-SEC16$^{1639-2195}$	4A	Yorimitsu and Sato, 2012
pGAD-Sec16$^{1856-2195}$	pGAD-C1	P_{ADH1}-AD-SEC16$^{1856-2195}$	4AC	This study
pGAD-Sec16$^{1968-2195}$	pGAD-C1	P_{ADH1}-AD-SEC16$^{1968-2195}$	4A	This study
pGAD-Sec16$^{1639-1967}$	pGAD-C1	P_{ADH1}-AD-SEC16$^{1639-1967}$	4A	Yorimitsu and Sato, 2012
pGAD-Sec16$^{1639-1996}$	pGAD-C1	P_{ADH1}-AD-SEC16$^{1639-1996}$	4A	This study
pGAD-Sec16$^{1639-2195\Delta1856-1967}$	pGAD-C1	P_{ADH1}-AD-SEC16$^{1639-2195\Delta1856-1967}$	4A	This study
pGDBU-Sec23	pGDBU-C1	P_{ADH1}-BD-SEC23	4A	Yorimitsu and Sato 2012
pGDBU-Sed4C	pGDBU-C1	P_{ADH1}-AD-SED4$^{1-346}$	4AC	This study
pGDBU-Sec12C	pGDBU-C1	P_{ADH1}-AD-SEC12$^{1-354}$	4AC	This study
pGDBU-Sec12C4b1-5	pGDBU-C1	P_{ADH1}-AD-SEC12$^{1-13}$-SED4$^{14-269}$-SEC12$^{275-354}$	4C	This study
pGDBU-Sec12C4b2-5	pGDBU-C1	P_{ADH1}-AD-SEC12$^{1-66}$-SED4$^{64-269}$-SEC12$^{275-354}$	4C	This study
pGDBU-Sec12C4b1-3	pGDBU-C1	P_{ADH1}-AD-SEC12$^{1-13}$-SED4$^{14-176}$-SEC12$^{182-354}$	4C	This study
pGDBU-Sec12C4b1,2	pGDBU-C1	P_{ADH1}-AD-SEC12$^{1-13}$-SED4$^{14-126}$-SEC12$^{132-354}$	4C	This study
pGDBU-Sec12C4b2,3	pGDBU-C1	P_{ADH1}-AD-SEC12$^{1-66}$-SED4$^{64-176}$-SEC12$^{182-354}$	4C	This study
pGDBU-Sec12C4b3-5	pGDBU-C1	P_{ADH1}-AD-SEC12$^{1-123}$-SED4$^{119-269}$-SEC12$^{275-354}$	4C	This study

ΔO-mannosylation mutations; T389A, T390A, S395A, S396A, S397A, T400A, T409A, T496A, S501A, T503A, S512A, S517A, S545A, S546A, S552A, S554A, T557A, T621A, S622A, S627A, S631A, T632A, T638A, T642A, T646A, S656A, S658A, S682A, S683A, T804A, T806A, S807A, S813A, T814A, S820A, S821A, S827A, S898A, S901A, S906A, T912A, S914A, S944A, S946A, S947A, S951A, S964A, S965A, S968A, S969A, S979A, S984A, T987A, T991A, T992A

Journal of Cell Science • Supplementary information
